# Dominant gut *Prevotella copri* in gastrectomised non-obese diabetic Goto–Kakizaki rats improves glucose homeostasis through enhanced FXR signalling

**DOI:** 10.1007/s00125-020-05122-7

**Published:** 2020-03-16

**Authors:** Noémie Péan, Aurelie Le Lay, Francois Brial, Jessica Wasserscheid, Claude Rouch, Mylène Vincent, Antonis Myridakis, Lyamine Hedjazi, Marc-Emmanuel Dumas, Elin Grundberg, Mark Lathrop, Christophe Magnan, Ken Dewar, Dominique Gauguier

**Affiliations:** 1 Inserm UMR 1124, Université de Paris, 45 rue des Saint-Pères, 75006 Paris, France; 2 McGill University and Genome Quebec Innovation Centre, 740 Doctor Penfield Avenue, Montreal, QC H3A 0G1 Canada; 3 Unit of Functional and Adaptive Biology, UMR 8251, CNRS, Université de Paris, 4 rue Marie Andrée Lagroua Weill-Halle, Paris, France; 4 Section of Biomolecular Medicine, Division of Computational and Systems Medicine, Department of Surgery and Cancer, Faculty of Medicine, Imperial College London, London, UK; 5 Beemetrix SAS, Massy, France

**Keywords:** 16S rDNA, Bile acids, Goto–Kakizaki rat, Microbiome, Type 2 diabetes

## Abstract

**Aims/hypothesis:**

Drug and surgical-based therapies in type 2 diabetes are associated with altered gut microbiota architecture. Here we investigated the role of the gut microbiome in improved glucose homeostasis following bariatric surgery.

**Methods:**

We carried out gut microbiome analyses in gastrectomised (by vertical sleeve gastrectomy [VSG]) rats of the Goto–Kakizaki (GK) non-obese model of spontaneously occurring type 2 diabetes, followed by physiological studies in the GK rat.

**Results:**

VSG in the GK rat led to permanent improvement of glucose tolerance associated with minor changes in the gut microbiome, mostly characterised by significant enrichment of caecal *Prevotella copri*. Gut microbiota enrichment with *P. copri* in GK rats through permissive antibiotic treatment, inoculation of gut microbiota isolated from gastrectomised GK rats, and direct inoculation of *P. copri*, resulted in significant improvement of glucose tolerance, independent of changes in body weight. Plasma bile acids were increased in GK rats following inoculation with *P. copri* and *P. copri*-enriched microbiota from VSG-treated rats; the inoculated GK rats then showed increased liver glycogen and upregulated expression of *Fxr* (also known as *Nr1h4*), *Srebf1c*, *Chrebp* (also known as *Mlxipl*) and *Il10* and downregulated expression of *Cyp7a1*.

**Conclusions:**

Our data underline the impact of intestinal *P. copri* on improved glucose homeostasis through enhanced bile acid metabolism and farnesoid X receptor (FXR) signalling, which may represent a promising opportunity for novel type 2 diabetes therapeutics.

**Electronic supplementary material:**

The online version of this article (10.1007/s00125-020-05122-7) contains peer-reviewed but unedited supplementary material, which is available to authorised users.

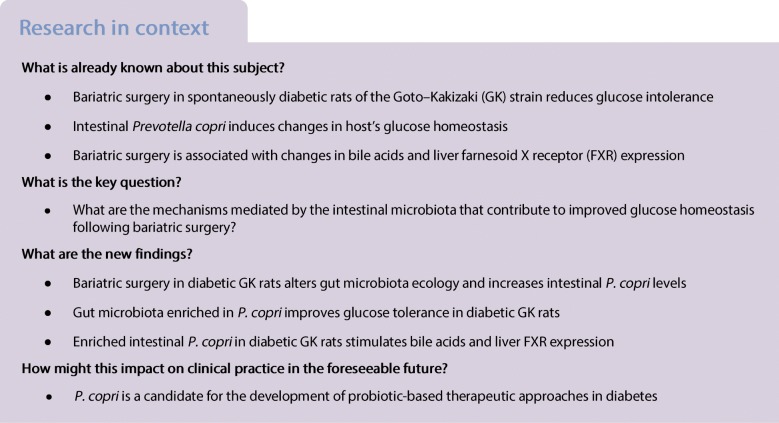



## Introduction

The gastrointestinal microbiota is a complex system of commensal bacteria which plays crucial roles in host metabolism [1] and maturation of the immune system [2]. Its architecture adapts to disease conditions [3], including obesity and type 2 diabetes [4, 5], and to diabetes therapies, such as drug treatment and bariatric surgery [6, 7]. Bariatric surgery techniques designed to induce weight loss in obese patients [8] are also associated with improved glucose homeostasis and even diabetes remission [9], but the underlying biological processes remain poorly understood. Alterations in the gut microbiome are proposed to mediate weight loss and improve glucose homeostasis following bariatric surgery [7].

Inbred preclinical models maintained in conditions limiting inter-individual phenotypic variability are powerful experimental systems to assessing functional relationships between gut microbiota and host physiology. The Goto–Kakizaki (GK) rat is an inbred model of spontaneous diabetes obtained by selective enrichment of naturally occurring genetic polymorphisms [10] resulting in multifaceted pathological features relevant to type 2 diabetes in the absence of obesity. Even though diabetes-causing genes have been mapped to the GK genome (reviewed in [11]) [12], bariatric surgery improves glycaemic control in this strain [13–15] through unknown mechanisms.

To disentangle relationships between gastrectomy and improved glucose homeostasis, we carried out a series of metagenomic and physiological experiments in the GK rat in order to test associations between *Prevotella copri*, a dominant bacterial strain stimulated by vertical sleeve gastrectomy (VSG), the metabolism of bile acids and farnesoid X receptor (FXR) signalling. These data bring new insights into the impact of gut microbiota on glycaemic control associated with enhanced bile acid metabolism and FXR signalling.

## Methods

For detailed Methods, please refer to the electronic supplementary material (ESM).

### Animals

**Fig. 1:**
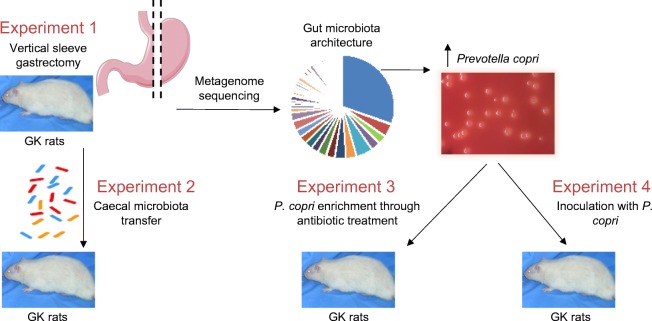
Outline of experimental design

Goto–Kakizaki (GK/Ox) rats bred in the Inserm UMR 1124 laboratory were maintained in a controlled environment (12 h dark–light cycles 22–24°C; humidity 50–60%). They had ad libitum access to water and standard chow (SAFE, Augy, France). Animals from the experimental and control groups were housed in different cages to avoid unintentional microbiota transplants via coprophagy. GK rats were used in a series of experiments outlined in Fig. 1. Procedures were reviewed by the University Ethics Committee in Animal Experiment and delivered by the French ministry of research under the licence 4231201602231507187.

### VSG

VSG was performed in 16-week-old male GK rats. The lateral 80% of the stomach was excised with a cutter (TLC55, Ethicon, Issy, France). Control GK rats were sham operated by application of pressure between the oesophageal sphincter and the pylorus. Rats were killed 13 weeks after surgery. Caecum and colon contents were harvested, quick-frozen and stored at  −80°C.

### Pair-feeding studies

Thirty days after surgery, sham rats were pair-fed to match the food intake of free-fed VSG GK rats. Blood glucose was determined using an Accu-Check, Performa (Roche Diagnostics, Meylan, France).

### Gut microbiota transfer

Five month old male GK rats were treated orally with Inexium (Omeprazole, AstraZeneca, Courbevoie, France) and Moviprep (Norgine, Rueil-Malmaison, France) [16, 17], and inoculated with caecal microbiota from VSG-treated or sham-operated GK rats.

### Enrichment of gut microbiota with *P. copri*

Five-month-old male GK rats were given vancomycin (0.5 g/l) and kanamycin (1 g/l) for 10 days. GK controls remained in antibiotic-free conditions.

### *P. copri* supplementation

Five-month-old male GK rats were given vancomycin (0.5 g/l), neomycin (1 g/l), metronidazole (1 g/l) and ampicillin (1 g/l) over 10 days to wipe out gut bacteria and facilitate *P. copri* colonisation. Then rats were inoculated with single gavage of 5 × 10^8^ CFU *P. copri* (DSM18205, DSMZ, Braunschweig, Germany) or heat-killed *P. copri* in controls.

### Glucose tolerance tests and sample collection and analysis

OGTTs (Experiment 1 in Fig. 1) and IPGTTs (Experiments 2, 3 and 4 in Fig. 1) were performed in conscious overnight fasted rats. Blood collected from the tail vein before administration of glucose (1 g/kg body weight) and sequentially afterwards was used to determine blood glucose (Roche Diagnostics) and insulin levels (Mercodia, Uppsala, Sweden).

Three days later, overnight fasted rats were killed. Plasma, adipose tissue, liver and caecal content were quick-frozen and stored at −80°C. Colorimetric assays were used to determine triacylglycerol (ab65336; Abcam, Paris, France) and glycogen (Sigma-Aldrich, Saint-Quentin, France) content.

### Metagenome sequencing

Bacterial DNA was prepared from caecal and colon samples (DNAStool mini kit, QIAGEN, Courtaboeuf, France). Whole-genome shotgun sequencing was performed on Illumina HiSeq 2000 (Illumina, San Diego, CA, USA). The sequence motifs corresponding to a 55 nucleotide segment of the bacterial 16S rRNA variable 1-3 region were used to estimate sample biodiversity.

### Quantitative PCR

*P. copri* enrichment was assessed by quantitative PCR of caecum bacterial DNA. Preparation of liver and adipose tissue RNA and quantitative RT-PCR were performed as described [18]. Oligonucleotide sequences are given in ESM Table 1.

### Quantitative analysis of plasma bile acids

Bile acids were quantified as described [19] using an ACQUITY BEH C8 column and a Xevo TQ-S mass spectrometer (both from Waters, Manchester, UK).

### Statistical analyses

R packages were used to assess differences in the frequency of 16S rDNA motifs. *p* values were corrected for multiple testing using the Benjamini–Hochberg method [20]. Blood glucose and insulin data during the glucose tolerance tests were analysed with the Kruskal–Wallis test. Non-parametric Mann–Whitney *U* tests were used to analyse physiological phenotypes, bile acid data and gene expression.

## Results

### Gastrectomised GK rats exhibit improved glycaemic control

**Fig. 2:**
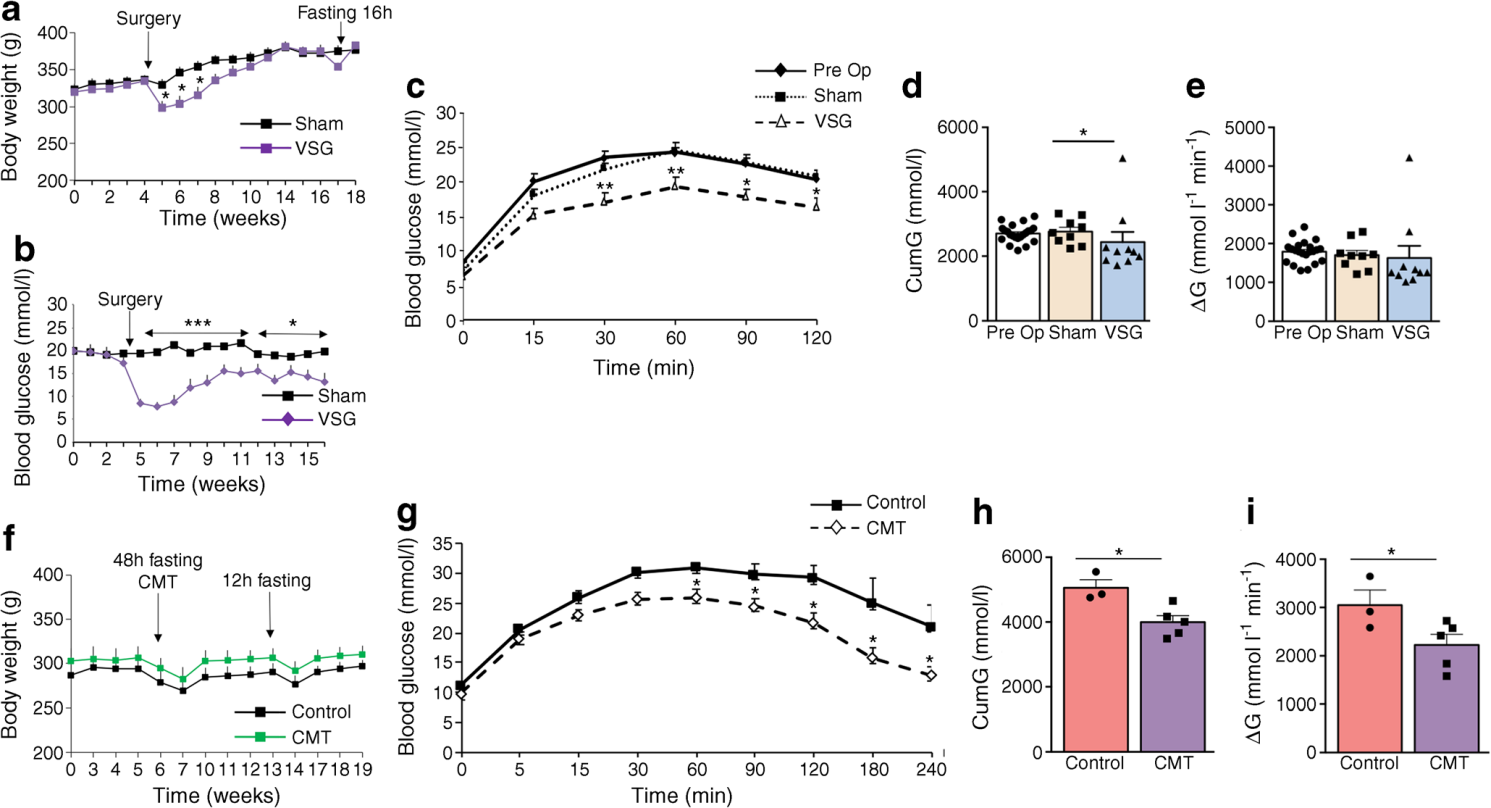
Effects of VSG and gut microbiota transfer in gastrectomised GK rats. Changes in body weight (**a**, **f**), blood glucose (**b**) and glucose tolerance (**c**–**e**; **g**–i) in GK rats following VSG or sham operation (**a**–**e**), and following inoculation of GK rats with gut microbiota from gastrectomised GK rats or sham controls (**f**–**i**). OGTTs (**c**) were performed following an overnight (16 h) fast, before VSG (Pre Op) and 91 days after VSG (*n* = 10) or sham operation (*n* = 9) (**c**–**e**). IPGTTs (**g**) were performed 12 days after caecal microbiota transfer (CMT) from VSG-treated GK rats (*n* = 5) or sham-operated GK rats (controls; *n* = 3–4) (**g**–**i**). Data are mean ± SEM. The Kruskal–Wallis test was used to analyse glucose tolerance data. The non-parametric Mann–Whitney *U* test was used to analyse the other variables. *p < 0.05, **p < 0.01, ***p < 0.001 vs sham-operated GK rats (**a**–**e**) or GK rats inoculated with microbiota from sham-operated GK rats (**g**–**i**). CumG, cumulative glucose levels during the glucose tolerance test; ΔG, cumulative glucose levels above baseline

To verify the effect of bariatric surgery on glycaemic control in the GK rats [13–15], we performed VSG in our GK colony (Experiment 1 in Fig. 1). VSG induced an immediate reduction in body weight (Fig. 2a) and food intake (ESM Fig. 1) and decreased glucose levels (Fig. 2b) when compared with sham-operated GK rats. Gastrectomised rats regained body weight similar to that of controls between 4 and 6 weeks post surgery, but blood glucose remained significantly reduced over the 12 week period following VSG (Fig. 2b), when OGTTs were performed.

Due to extensive development of intra-abdominal connective tissue secondary to VSG, OGTTs were preferred to IPGTTs. Glucose tolerance was similar in GK rats before VSG and sham-operated GK, thus demonstrating that glucose homeostasis was not affected by sham operation (Fig. 2c). VSG resulted in improved glucose tolerance (Fig. 2c), assessed by the significant reduction of both cumulative glucose levels during the OGTT (Fig. 2d) and the ΔG parameter (Fig. 2e) when compared with sham GK rats. Gastrectomised GK rats exhibited elevated basal insulin levels and enhanced glucose-induced insulin secretion when compared with controls (ESM Fig. 2a).

### Inoculation of GK rats with gut microbiota from gastrectomised GK rats improves glucose tolerance

To test whether the gut microbiota may mediate the metabolic effects of VSG, we inoculated GK rats with the caecal microbiota from gastrectomised or sham-operated GK rats (Experiment 2 in Fig. 1). Body weight was affected neither by antibiotic treatment prior to microbiota transplantation nor by gut microbiota transfer (Fig. 2f). Blood glucose was significantly reduced from 60 min after the glucose injection until the end of the IPGTT in rats inoculated with the microbiota from VSG-treated rats (Fig. 2g), which resulted in a significant reduction in both cumulative glucose levels (Fig. 2h) and the ΔG parameter (Fig. 2i) when compared with GK rats inoculated with the microbiota from sham-operated animals. There were no differences in insulin secretion in the two groups (ESM Fig. 2b). These data demonstrate that gut microbiota changes induced by VSG in diabetic GK rats contribute to improving glucose tolerance when transplanted to diabetic recipients.

### VSG and dietary restriction are equally efficient to reduce hyperglycaemia in GK rats

**Fig. 3:**
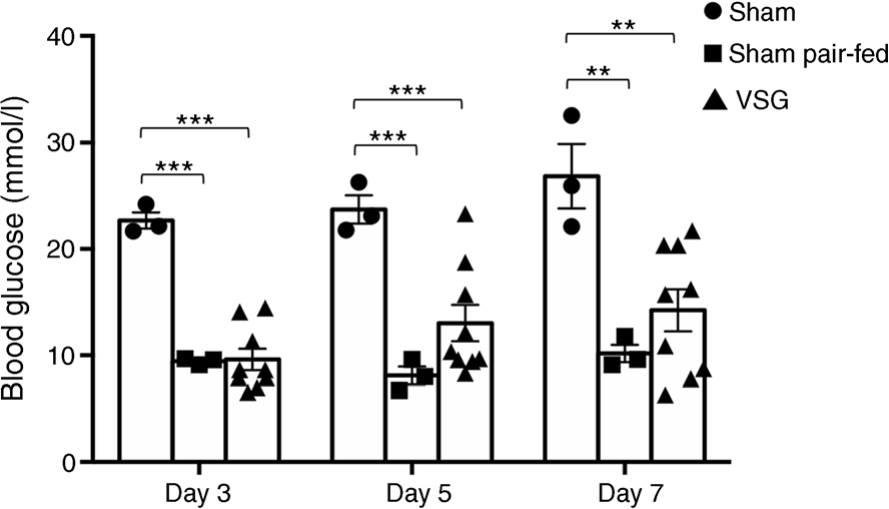
Impact of caloric restriction on blood glucose in sham-operated GK rats. Blood glucose was determined ad libitum in a group of sham-operated GK rats (*n* = 3) 3, 5 and 7 days after the beginning of the pair-feeding experiment and compared with free-fed sham-operated (*n* = 3) and gastrectomised (VSG; *n* = 9) GK rats. Non-parametric Mann–Whitney *U* tests were applied for statistical data analysis. **p < 0.01, ***p < 0.001 vs sham-operated GK rats

To evaluate the performance of VSG in improving glucose homeostasis in the GK strain, we carried out a pair-feeding experiment in GK rats 30 days after the sham operation. Blood glucose was systematically significantly reduced in caloric restricted sham-operated GK rats when compared with free-fed sham-operated GK rats (Fig. 3). Interestingly, the magnitude of the drop in glucose levels following caloric restriction in sham-operated GK rats and VSG in GK rats was very similar, demonstrating the efficiency of VSG in improving glucose homeostasis.

### VSG alters gut microbiota architecture

To identify changes in the gut microbiota architecture that may explain VSG-induced improvement in glucose homeostasis, we carried out metagenome sequencing in gastrectomised and sham-operated GK rats. We sequenced bacterial DNA from caecum and colon content collected 14 weeks after VSG when glucose intolerance was reduced in gastrectomised GK rats, whilst body weight returned to control values (Table 1). These intestinal sites are the richest in bacteria and most active for nutrient absorption. The 27 microbiota samples generated a consistent amount of sequencing data ranging from 21.6 Gb per sample in the colon of VSG-treated rats to 22.9 Gb in the caecum of control rats (Table 1). Considering the high technical reproducibility of each sample across the four sequencing runs (ESM Fig. 3) data from technical replicates were pooled.

**Table 1:** Details of metagenome sequencing of caecum and colon samples from gastrectomised and sham-operated GK rats

	VSG		Sham	
Intestinal site	Caecum (10)	Colon (6)	Caecum (8)	Colon (3)
Mean length (Gb)	22.4 ± 3.7	21.6 ± 3.6	22.9 ± 3.1	21.8 ± 1.1
Total rDNA motifs	16,253 ± 3940	18,485 ± 6478	16,138 ± 3671	16,067 ± 1089
Unique rDNA motifs	1302 ± 156	1281 ± 170	1367 ± 167	1427 ± 89
Frequency >5%	1.25 (38.38)	0.31 (28.13)	1.25 (27.98)	0.32 (8.00)
Frequency 0.5–5%	8.10 (35.54)	9.97 (52.57)	10.28 (39.18)	11.21 (55.49)
Frequency <0.5%	90.65 (23.01)	89.72 (18.78)	88.47 (23.98)	88.47 (19.29)

Data are mean ± SD or frequency (proportion)

The number of biological replicates for each treatment group and intestinal site is given in parentheses

The total amount of sequencing data obtained (mean length, in Gb) is given. Similar numbers of total and unique rDNA motifs were found in the four groups

The proportion of highly frequent (>5%), moderately frequent (0.5–5%) and rare (<0.5%) motifs was calculated. The proportion of the gut microbiome that these categories account for is given in parentheses

The depth of sequencing allowed for over 16,000 observations of rDNA motifs per caecum and colon sample (Table 1) thus providing confidence for detecting any bacterial species present at 1:5000 or better. Over 1200 unique rDNA motifs were identified in each sample (range 1281–1427) (Table 1). Overall, sequencing data in all samples and all intestinal sites identified 8767 unique rDNA motifs (517,697 occurrences).

We used these data to assess sequencing data consistency in the caecum and colon within individual animals and subsequently caecum–colon, caecum–caecum and colon–colon between animals of the same group. Mean and SD of the 100 most common 16S rDNA sequences illustrate the relatively large variability of data from the most frequent motifs in caecum and colon (ESM Figs 4a, b and 5a, b). Data from caecum and colon from the same animal were generally more similar (up to 99%) than any combination from different animals (ESM Figs 4c and 5c). For samples with paired caecum–colon microbiome sequencing datasets, comparisons of the 10 most prevalent rDNA motifs across all samples illustrate that within-individual similarity is higher than between-individual similarity (ESM Fig. 6).

**Fig. 4:**
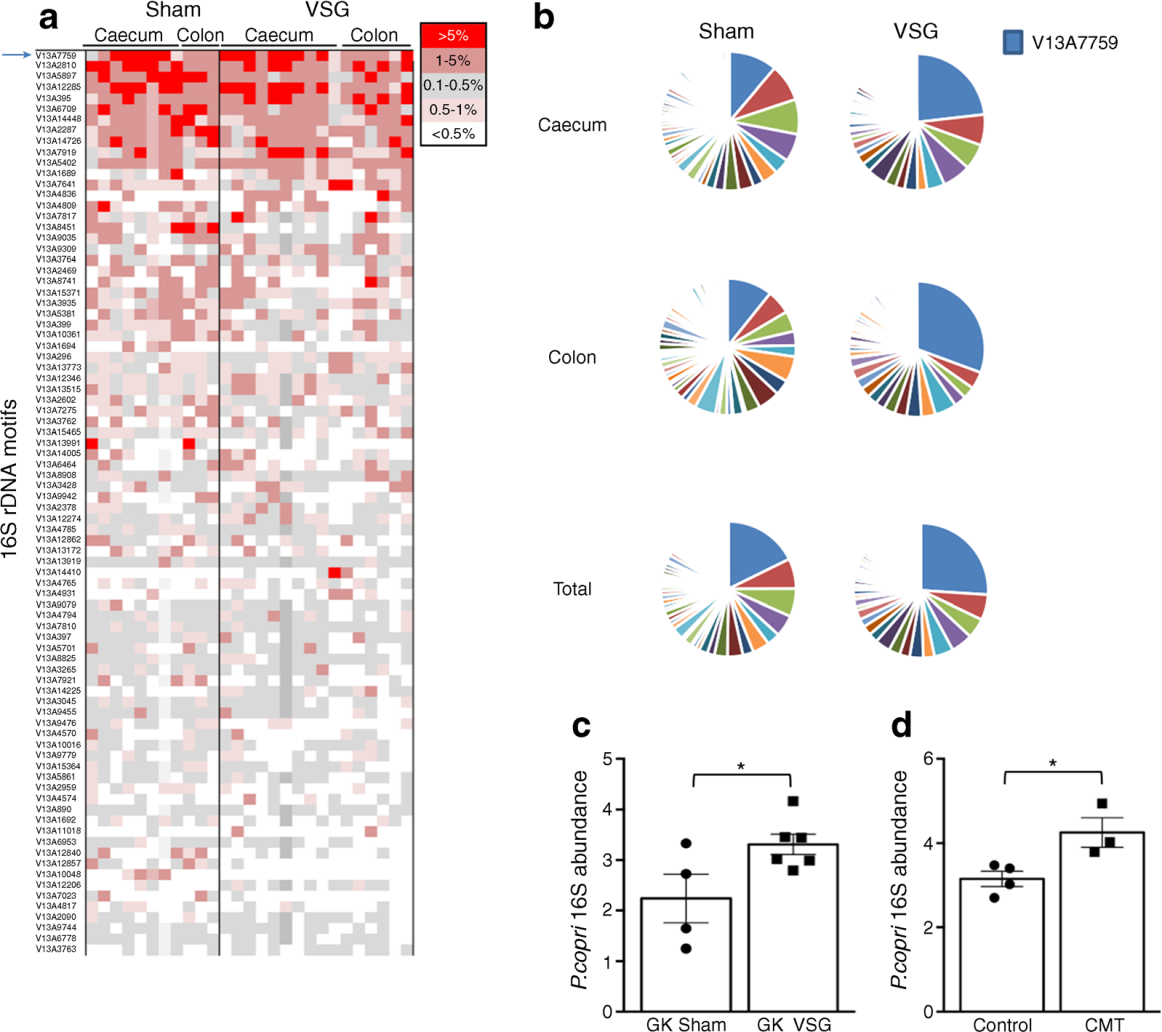
Effects of VSG on gut microbiome architecture in the GK rat. Frequency of 16S rDNA motifs derived from metagenome sequencing was calculated in each caecum and colon sample from GK rats following VSG (*n* = 10) or sham operation (*n* = 8) (**a**). Means of rDNA motif frequencies were calculated for data from caecum, colon and caecum and colon combined in the two rat groups (**b**). Data are shown for the 84 most abundant motifs. Each line in (**a**) represents a different motif colour-coded according to its frequency and each colour in (**b**) represents the relative proportion of each motif; the arrow in (**a**) and the blue colour in (**b**) represent V13A7759. Quantitative RT-PCR was carried out to assess enrichment of the motif V13A7759 (*P. copri*) in GK rats following VSG (*n* = 6) (**c**) and GK rats following caecal microbiota transfer (CMT) from VSG-treated GK rats (*n* = 3) (**d**); *n* = 4 for sham/control in both experiments. Details of all rDNA motifs, associated frequencies and statistical differences between the rat groups are given in ESM Table 2. Data are mean ± SEM. Non-parametric Mann–Whitney *U* tests were applied for statistical analysis of *P. copri* abundance. *p < 0.05 vs sham-operated GK rats (**c**) or GK rats inoculated with microbiota from sham-operated GK rats (**d**)

We carried out exact match searches for a 55mer sequence beginning with the sequence of the V13A reverse primer and selected 232 rDNA motifs present at an occurrence of >0.005% in at least 50% of individuals, regardless of the sample’s intestinal origin (ESM Table 2). Despite the large numbers of unique rDNA motifs identified in the dataset, the 52 most prevalent rDNA motifs accounted for 80% of the total data. Given the depth of metagenome sequencing and extensive within-individual similarities in rDNA motifs in caecum and colon, caecal sequence data were used in analyses of 16S rDNA motif enrichment. Each individual microbiome was dominated by few rDNA present at a frequency >5% in gastrectomised and control rats (Fig. 4a, b, ESM Table 2, ESM Fig. 7), representing only 0.31–1.25% of the motifs, but accounting for a large proportion of the overall motif abundance (up to 38.38% in the caecum of gastrectomised rats) (Table 1). In contrast, the largest diversity of the microbiome, representing over 88% of distinct motifs, was accounted for by motifs present at very low frequency (<0.5%) and collectively covering only 18.78–23.98% of the sequenced microbiome (Table 1).

### *P. copri* dominates GK gut microbiota and is enriched by VSG

We next searched for differentially enriched rDNA motifs in gastrectomised and control rats. We identified ten motifs over-represented in VSG-treated rats and 16 motifs over-represented in controls (Table 2). Two motifs were present only in the gut microbiome of sham (V13A9832) or VSG (V13A7019) rats. The most frequent rDNA motif (V13A7759), accounting for up to 54.5% of the microbiome of individual samples, was significantly over-represented in gastrectomised GK rats (22.71%) than in controls (9.07%, *p* = 0.046) (Table 2, Fig. 4b). Sequence homology searches in microbial genome databases identified only 9 rDNA motifs showing maximum sequence similarities (100%) with the DNA sequence of a single (V13A7759, V13A9569, V13A12536, V13A6709, V13A399, V13A234, V13A622) or several (V13A1861, V13A12857) bacteria (Table 2). The most predominant motif in GK rats (V13A7759) is homologous to scaffold assemblies of *P. copri* (Table 2). Its significant enrichment in gastrectomised GK rats and after inoculation of microbiota from gastrectomised GK rats was confirmed by quantitative PCR of caecum DNA (Fig. 4c, d).

**Table 2:** Frequency of rDNA motifs significantly enriched in the gut microbiota from gastrectomised (A) or sham-operated (B) GK rats

	Motif	Sham	VSG	*p* value	Sequence homology with bacteria	Accession
A (%)	V13A7759	9.074 ± 3.114	22.706 ± 5.539	0.046	*Prevotella copri* DSM 18205	NZ_GG703862
	V13A9309	0.350 ± 0.243	1.017 ± 0.228	0.015	–	–
	V13A14410	0.003 ± 0.002	0.636 ± 0.548	0.002	–	–
	V13A14225	0.064 ± 0.047	0.310 ± 0.097	0.020	–	–
	V13A12206	0.142 ± 0.116	0.206 ± 0.053	0.036	–	–
	V13A1861	0.031 ± 0.008	0.124 ± 0.024	0.002	*Mannheimia haemolytica* M42548	NC_021082
					*Mannheimia succiniciproducens* MBEL55E	NC_006300
					*Necropsobacter massiliensis* strain FF6	NZ_CDON01000005
					*Pasteurella bettyae* CCUG 2042	NZ_AJSX01000044
					*Rodentibacter heylii* strain 1,998,236,014	NZ_MLAA01000050
	V13A11382	0.010 ± 0.010	0.088 ± 0.039	0.014	–	–
	V13A9569	0.012 ± 0.003	0.035 ± 0.007	0.008	*Anaerotignum lactatifermentans* DSM 14214	NZ_FRAH01000002
	V13A12536	0.001 ± 0.001	0.020 ± 0.008	0.018	*Planococcus halocryophilus* Or1	NZ_CP016537
	V13A7019	Absent	0.008 ± 0.004	0.020	–	–
B (%)	V13A6709	3.043 ± 0.538	1.494 ± 0.237	0.046	*Prevotella pleuritidis* JCM 14110	NZ_BAJN01000030
	V13A8451	1.814 ± 0.615	0.096 ± 0.040	0.008	–	–
	V13A399	0.902 ± 0.117	0.484 ± 0.167	0.011	*Planktothrix agardhii* NIVA-CYA 126/8	NZ_CM002803
	V13A10048	0.665 ± 0.266	0.004 ± 0.003	0.033	–	–
	V13A12857	0.286 ± 0.141	0.023 ± 0.006	0.014	*Eubacterium eligens* ATCC 27750	NC_012778
					*Eubacterium hallii* DSM 3353	NZ_ACEP01000116
					*Eubacterium ramulus* ATCC 29099	NZ_KI271105
					*Lactobacillus rogosae* strain ATCC 27753	NZ_FONU01000001
	V13A9832	0.273 ± 0.097	Absent	0.021	–	–
	V13A234	0.148 ± 0.031	0.071 ± 0.058	0.019	*Bacteroides paurosaccharolyticus* JCM 15092	NZ_BAJR01000054
	V13A686	0.137 ± 0.052	0.022 ± 0.011	0.036	–	–
	V13A603	0.136 ± 0.057	0.018 ± 0.018	0.037	–	–
	V13A7033	0.130 ± 0.019	0.065 ± 0.009	0.011	–	–
	V13A622	0.075 ± 0.036	0.015 ± 0.005	0.048	*Ruminococcus flavefaciens* MC2020	NZ_JNKE01000007
	V13A1295	0.073 ± 0.015	0.035 ± 0.011	0.046	–	–
	V13A6720	0.032 ± 0.013	0.005 ± 0.003	0.019	–	–
	V13A1442	0.024 ± 0.006	0.001 ± 0.001	0.002	–	–
	V13A6490	0.018 ± 0.007	0.004 ± 0.002	0.012	–	–
	V13A12243	0.011 ± 0.004	0.002 ± 0.001	0.025	–	–

Data are shown as means of motif frequency ± SEM

Sequences of the rDNA motifs are given in ESM Table 2, along with data from the set of rDNA motifs present in caecum samples in at least 50% of individual rats at a frequency >0.005%. Only known bacteria showing full sequence homology with rDNA motifs are given

### Intestinal *P. copri* enrichment improves glucose regulation in GK rats

**Fig. 5:**
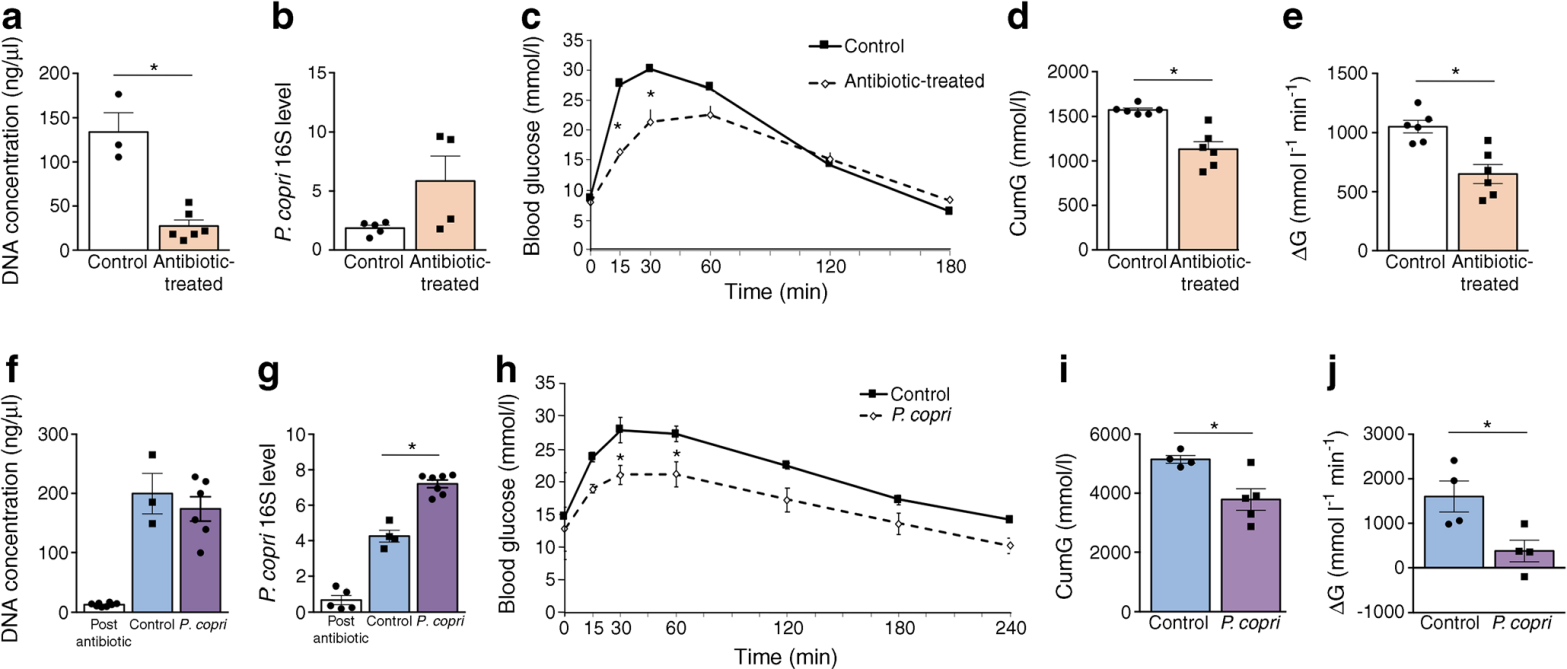
Effects of gut microbiota enrichment in *P. copri* on glucose homeostasis in GK rats. GK rats were either treated with antibiotics permissive to *P. copri* or remained in antibiotic-free conditions (**a**–**e**). A separate group of GK rats was inoculated with *P. copri* or heat-inactivated *P. copri* (**f**–**j**). Faecal DNA concentration and *P. copri* abundance were determined in antibiotic-treated (*n* = 3–6) and control (*n* = 4–6) rats (**a**, **b**). Faecal DNA concentration and *P. copri* abundance were determined in GK rats treated with a combination of broad spectrum antibiotics prior to inoculation (post antibiotics) (*n* = 5), and in rats inoculated with *P. copri* (*n* = 6) or heat-inactivated *P. copri* (control) (*n* = 3–4) (**f**, **g**). Glucose tolerance (**c**–**e**, **h**–**j**) was determined following an IPGTT in overnight fasted (16 h) GK rats 10 days after antibiotic treatment or *P. copri* inoculation. Data are mean ± SEM. The Kruskal–Wallis test was used to analyse glucose tolerance data. The non-parametric Mann–Whitney *U* test was used to analyse the other variables. *p < 0.05 vs control. CumG, cumulative glucose levels during the IPGTT; ΔG, cumulative glucose levels above baseline

Owing to the controversial role of *P. copri* on cardiometabolic phenotypes [21, 22], we focused functional studies on this bacterial species, which was one of the few commercially available species differentially represented in our model systems. To test the hypothesis of a role of *P. copri* on improved glucose regulation, we treated GK rats with antibiotics (vancomycin and kanamycin) permitting its growth and resulting in *P. copri* intestinal enrichment [23] (Experiment 3 in Fig. 1).We verified that antibiotics significantly reduced bacterial DNA concentration in faeces (Fig. 5a), thus inhibiting the gut bacterial ecosystem, and that it significantly increased the proportion of *P. copri* (Fig. 5b). Antibiotics did not affect body weight (ESM Fig. 8a), but significantly improved glucose tolerance. Antibiotic treatment resulted in significant reduction in blood glucose 15 and 30 min after intraperitoneal glucose injection (Fig. 5c), in cumulative blood glucose (Fig. 5d) and in the ΔG parameter (Fig. 5e) when compared with untreated GK controls. Glucose-induced insulin secretion was similar in the two rat groups (ESM Fig. 9a).

### *P. copri* inoculation improves glucose regulation in GK rats

To further assess the impact of *P. copri* enrichment on glucose homeostasis, we inoculated GK rats with 5 × 10^8^ CFU *P. copri* or with heat-killed *P. copri* (Experiment 4 in Fig. 1). We initially measured faecal DNA concentration to verify microbiota abolition by the antibiotics. We then verified by PCR that gut microbiota enrichment in *P. copri* was implemented 12 days after bacterial inoculation (Fig. 5f, g). *P. copri* treatment had no effect on body weight (ESM Fig. 8b), but was associated with a significant reduction of glucose intolerance when compared with GK rats treated with heat-inactivated *P. copri*. Blood glucose after intraperitoneal glucose injection was lower in *P. copri*-treated GK rats than in controls (Fig. 5h). *P. copri* inoculation resulted in a significant reduction in cumulative blood glucose levels during the glucose tolerance test (Fig. 5i) and in the ΔG parameter (Fig. 5j). *P. copri* had no significant impact on insulin secretion (ESM Fig. 9b).

Our data demonstrate that increased representation of intestinal *P. copri* in GK rats improves glucose homeostasis.

### A gut microbiota enriched in *P. copri* stimulates bile acid metabolism

**Fig. 6:**
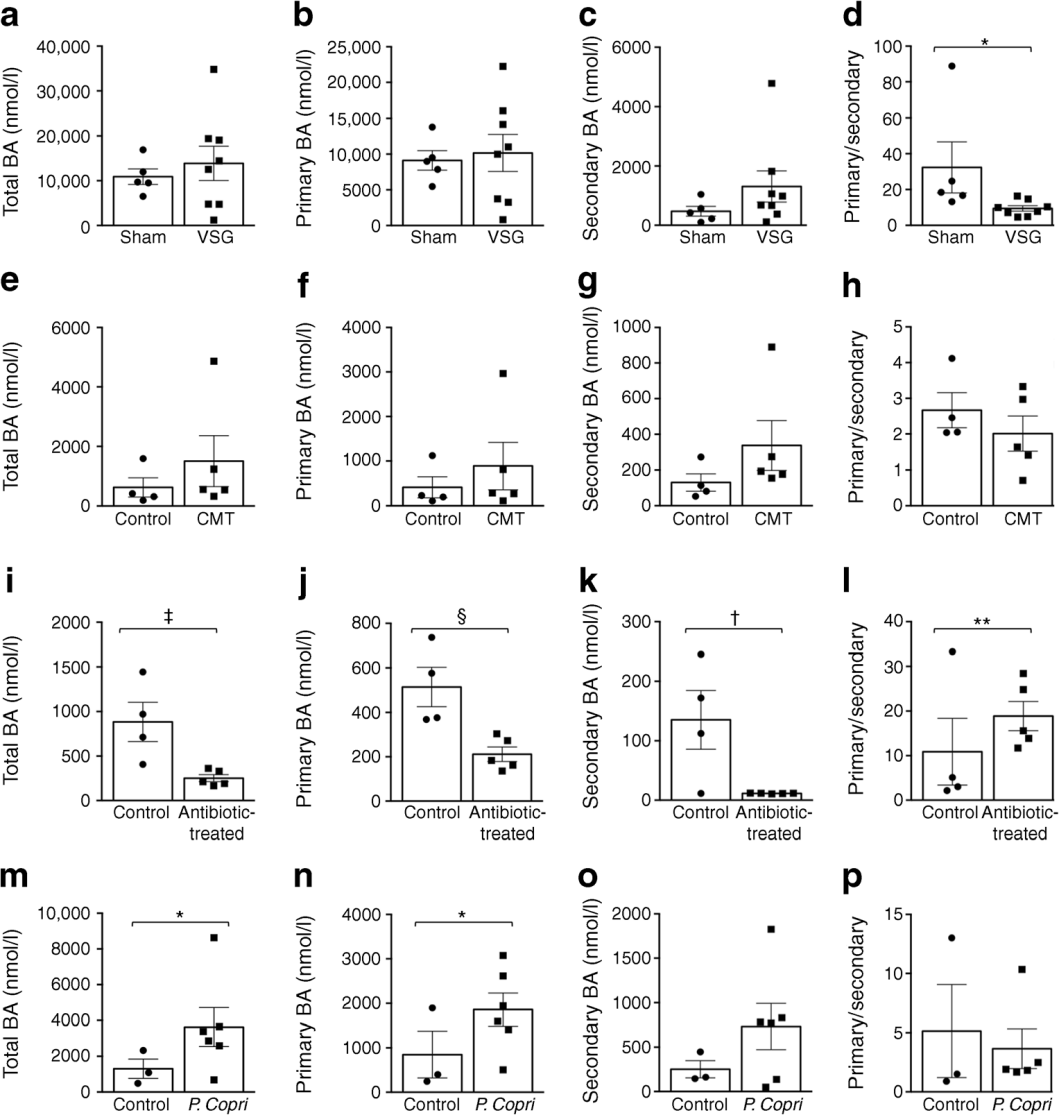
Effects of intestinal *P. copri* enrichment on plasma bile acid concentrations in the GK rat. Mass spectrometry methods were used to determine the plasma concentration of bile acids in GK rats following VSG (*n* = 8) or sham operation (*n* = 5) (**a**–**d**), in GK rats following caecal microbiota transfer (CMT) from VSG-treated GK rats (*n* = 5) or sham-operated GK rats (*n* = 4) (**e**–**h**), in GK rats treated with a combination of kanamycin and vancomycin antibiotics (*n* = 5) or in the absence of antibiotics in controls (*n* = 4) (**i**–**l**), or in GK rats inoculated with *P. copri* (*n* = 6) or heat-inactivated *P. copri* (*n* = 3) (**m**–**p**). Non-parametric Mann–Whitney *U* tests were used for statistical analysis. Data are mean ± SEM. *p < 0.05, **p < 0.01, ^†^*p* = 0.05, ^‡^*p* = 0.06, ^§^*p* = 0.07 vs the relevant control. BA, bile acid

Given the consistent effects of bariatric surgery on improved glucose tolerance and increased circulating bile acids in humans [24], we hypothesised that *P. copri* intestinal enrichment in GK rats stimulates bile acid metabolism. Mass spectrometry allowed the quantification of 27–35 bile acids in plasma from all rat groups, which were analysed as individual molecules (ESM Tables 3 and 4) and total, primary and secondary bile acids (Fig. 6). VSG in GK rats significantly increased concentrations of ursocholanic, tauro-ursodeoxycholic (5β-cholanic acid-3α,7β-diol *N*-(2-sulphoethyl)) and taurohyodeoxycholic (5β-cholanic acid-3α,6α-diol *N*-(2-sulphoethyl)-amide) acids (ESM Table 3). Total and primary bile acid concentrations were similar in gastrectomised and sham-operated rats (Fig. 6a, b). The level of secondary bile acids, particularly deoxycholic acid, showed a 2.8-fold increase in VSG rats leading to a significant decrease in the ratio primary to secondary bile acids in gastrectomised rats (*p* = 0.030).

Likewise, caecal microbiota transfer from gastrectomised GK to GK rats was associated with significantly increased concentrations of ursocholanic, tauro-ursocholanic (5β-cholanic acid *N*-(2-sulphoethyl)-amide)) and isolithocholic acids (ESM Table 4). Treated GK rats showed a marked stimulation of total (+239%), primary (+214%) and secondary (+260%) bile acids (Fig. 6e–g). In contrast, many bile acids were downregulated by *P. copri* permissive antibiotics in GK rats, resulting in the downregulation of total, primary and secondary bile acids, and a 5.5-fold increase in the ratio of primary to secondary bile acids (*p* = 0.007) (ESM Table 4) (Fig. 6i–k). Finally, *P. copri* inoculation in GK rats increased the concentrations of many bile acids, including cholic (+518%, *p* = 0.008), allolithocholic (+454%, *p* = 0.016), chenodeoxycholic (+341%, *p* = 0.048) and ω-muricholic (+810%, *p* = 0.027) acids, resulting in significantly increased total (+460%, *p* = 0.047) and primary (+492%, *p* = 0.010) bile acids (ESM Table 4; Fig. 6m, n).

Overall, bile acids were stimulated upon intestinal enrichment of *P. copri*. Interestingly *P. copri* inoculation was associated with increased levels of primary bile acids chenodeoxycholic acid (+341%, *p* = 0.048) and cholic acid (+610%, *p* = 0.009) (ESM Table 4), which activate FXR [24, 25].

### Enhanced liver expression of FXR is associated with stimulation of bile acid metabolism and improved glucose tolerance consecutive to intestinal *P. copri* enrichment

**Fig. 7:**
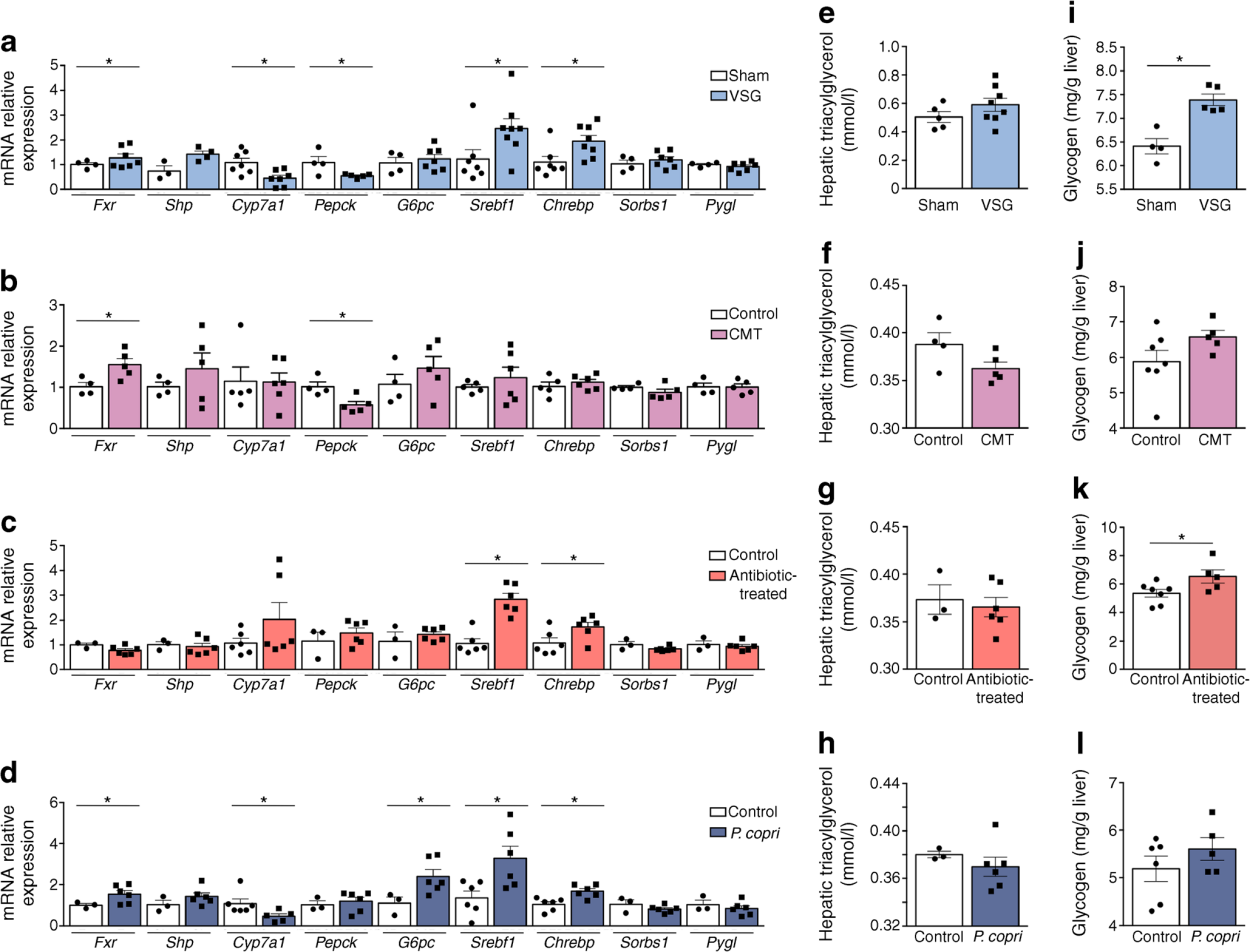
Liver gene expression and hepatic triacylglycerol and glycogen storage in treated GK rats and controls. Liver gene expression (**a**–**d**) assessed by quantitative RT-PCR and quantification of hepatic triacylglycerol content (**e**–**h**) and glycogen storage (**i**–**l**) were determined in GK rats following VSG (**a**, **e**, **i**), in GK rats following caecal microbiota transfer (CMT) from VSG-treated GK rats (**b**, **f**, **j**), in GK rats following treatment with *P. copri* permissive antibiotics (**c**, **g**, **k**) and in GK rats following inoculation with *P. copri* (**d**, **h**, **l**). *n* = 4–10 per group. Data are mean ± SEM. Non-parametric Mann–Whitney *U* tests were used for statistical analysis. *p < 0.05 vs sham/control rats

FXR activation has been consistently reported in response to bariatric surgery and associated stimulation of circulating bile acids [26]. We analysed the expression of *Fxr* (also known as *Nr1h4*) and genes relevant to its function in liver and adipose tissue of the four models generated in our study. Liver expression of *Fxr* was significantly upregulated in gastrectomised GK rats and in GK rats inoculated with gut microbiota from VSG GK rats or with *P. copri*, when compared with corresponding controls (Fig. 7a–d). A primary target of FXR (*Shp* [also known as *Nr0b2*]) and *G6pc*, which encodes glucose 6 phosphatase exhibited direction of expression changes similar to those of *Fxr* in the four rat groups. Expression of *Pepck* (encoding phosphoenolpyruvate carboxykinase) and *Cyp7a1* (encoding cholesterol 7α-hydroxylase [CYP7A1]/cytochrome P450), which are normally downregulated by bile acids and FXR, were inhibited in gastrectomised GK rats (*Pepck* and *Cyp7a1*), in GK rats treated with the gut flora of VSG GK rats (*Pepck*) and in GK rats inoculated with *P. copri* (*Cyp7a1*). Expression of the transcription factors *Chrebp* (also known as *Mlxipl*) and *Srebf1*, which regulate liver lipogenesis and cholesterol biogenesis, was significantly stimulated in VSG rats, in GK rats treated with *P. copri* permissive antibiotics and in GK rats inoculated with *P. copri* (Fig. 7a, c, d). Expression of *Sorbs1*, which is involved in insulin signalling, and *Pygl*, which encodes glycogen phosphorylase, was unaffected by VSG and by increased intestinal *P. copri* levels.

To test the functional relevance of stimulated *Fxr* transcription in the rat models, we analysed the hepatic concentration of triacylglycerol (Fig. 7e–h) and glycogen (Fig. 7i–l), which are both regulated by FXR. Triacylglycerol levels were unchanged in the experimental groups (Fig. 7e–h). In contrast, hepatic glycogen content was significantly more elevated in gastrectomised GK rats (Fig. 7i) and in GK rats treated with the *P. copri* permissive antibiotics (Fig. 7k) than in corresponding controls.

Finally, considering the functional consequences of bariatric surgery and FXR activation on adipose tissue metabolism and inflammation [27, 28], we analysed the expression of relevant genes in the adipose tissue in GK rats in response to VSG and enhanced intestinal *P. copri* (ESM Fig. 10). Consistent with the liver data, *Fxr* and *Srebf1* expression was co-ordinately upregulated in adipose tissue following VSG (ESM Fig. 10a). Treatment with *P. copri* permissive antibiotics and inoculation of *P. copri* in GK rats also resulted in stimulation of *Fxr* and *Srebf1* expression (ESM Fig. 10c, d). Expression of *Tnfa* was unchanged in all four experimental groups. In contrast, downregulated expression of *Il6* in GK rats inoculated with the microbiota of gastrectomised GK rats (ESM Fig. 10b) or with *P. copri* (ESM Fig. 10d) and upregulated expression of *Il10* in all four rat models (ESM Fig. 10a–d), suggest anti-inflammatory effects of *P. copri*.

Collectively, these data underline the role of intestinal *P. copri* in transcriptional regulation of FXR signalling, which contributes to improve glucose and lipid homeostasis.

## Discussion

Our data underline the long term effects of VSG on gut microbiota architecture remodelling and the reduction of glucose intolerance in the non-obese GK rat model of polygenic type 2 diabetes, which therefore avoids the confounding effects of weight loss induced by gastrectomy in individuals with both diabetes and obesity. Our data suggest that VSG may be equivalent, or even superior, to caloric restriction, which often results in only transient improvement in glucose homeostasis in diabetic patients [29]. Our results contribute to our understanding of the relationship between gastrectomy and improved glucose homeostasis, by demonstrating the involvement of a specific bacterial species (*P. copri*), which is stimulated by VSG, in the regulation of glucose tolerance and the activation of FXR and bile acid metabolism in the GK strain.

A causal relationship between cardiometabolic diseases and gastrointestinal microbiota has been inferred through associations between reduced disease risk and increased bacteria diversity and gene richness [30, 31] and the identification of bacteria associated with surgically-induced remission of diabetes in obese patients [32] and in preclinical models [33–36]. The most abundant rDNA motif enriched in gastrectomised GK rats aligned to the draft genome assembly of *P. copri.* Considering the dominance of *P. copri* in both GK and gastrectomised GK rats, gene richness is unlikely to significantly account for improved glucose homeostasis following VSG. On the other hand, our findings in GK rats carrying enriched intestinal *P. copri* abundance support the causal relationship between intestinal *P. copri* and improved glucose metabolism.

Investigations into the pathophysiological role of *P. copri* have led to apparently contradictory results, which may be due to host diet-dependent effects and the existence of several *P. copri* clades [37]. Intervention studies have demonstrated that faecal levels of *Prevotella* correlate with high fibre and high carbohydrate dietary intake [38–40]. High fibre diet improves glucose homeostasis and increases faecal *P. copri* levels, resulting in increased liver glycogen in mice inoculated with human microbiota enriched in *P. copri* [22]. On the other hand, *P. copri* is correlated with insulin resistance in humans and in fat-fed mice [21]. Faecal *P. copri* is elevated in type 2 diabetes [41]. Improved glucose tolerance and increased circulating bile acids in GK rats following intestinal *P. copri* enrichment concur with its beneficial metabolic role. We hypothesise that naturally increased intestinal *P. copri* in the GK rat contributes to the complex aetiology of diabetes in this strain by counteracting the adverse effects of permanent hyperglycaemia, along with sequence variants in genes that stimulate insulin secretion [12] and downregulated expression of genes involved in heart failure [42].

Changes in plasma bile acid composition, combined with altered gut microbiota ecology, are hallmarks of the adaptation to bariatric surgery in humans and in preclinical models [32, 43]. Activation of FXR by bile acids stimulates the expression of the nuclear receptor SHP, which downregulates hepatic CYP7A1, the key enzyme in bile acid biosynthesis that converts cholesterol to 7α-hydroxycholesterol [26, 44]. Given the impact of bariatric surgery on bile acid metabolism and FXR signalling [45] and the association between increased *P. copri* abundance and glucose metabolism [22], we reasoned that improved glucose tolerance in response to increased intestinal *P. copri* abundance in GK rats involves FXR signalling. Upregulated expression of *Fxr* and *Shp* and downregulated expression of *Cyp7a1*, combined with increased bile acid synthesis and experimentally induced gut microbial enrichment in *P. copri*, supports our hypothesis.

Paralleled overexpression of *Fxr* and the transcription factors *Chrebp* and *Srebp1c* (also known as *Srebf1*), which regulate the metabolism of triacylglycerols, fatty acids and phospholipids [46, 47], in both VSG-treated rats and GK rats inoculated with *P. copri*, may account for increased hepatic glycogen content identified in these models. Consistent overexpression of *Fxr* and *Srebp1c* in adipose tissue of gastrectomised GK rats and in our models of *P. copri* enrichment was associated with systematic increase in *Il10* expression, suggesting activation of anti-inflammatory mechanisms.

Our findings warrant further investigation. Bacterial species present at low frequency in the GK gut, but significantly differentially enriched following VSG, may contribute to improve glucose homeostasis, either individually or as part of bacterial ecosystems. Conversely, antibiotic treatment increasing intestinal *P. copri* abundance may lead to coordinated changes in the abundance of bacterial species that can also regulate glucose tolerance, and remain to be identified. In addition, other molecular systems, including gut hormones, may also account for restored glucose homeostasis in gastrectomised GK rats. For example, Roux-en-Y gastric bypass (RYGB) in GK rats enhances insulin secretion and suppresses glucagon secretion, and restores islet structure through stimulation of the peptide tyrosine (PYY) [48].

### Conclusions

Our results illustrate interactions between the gut microbiome and host metabolism following surgical therapy for diabetes. We demonstrate that *P. copri* is the dominant intestinal bacterial species in the GK non-obese model of polygenic type 2 diabetes, which is further stimulated by gastrectomy, and that its intestinal enrichment mimics the effects of bariatric surgery on both elevated circulating bile acids and stimulated expression of FXR to reduce glucose intolerance. *P. copri* represents an attractive candidate for the development of probiotic-based therapies in diabetes. The exact genomic structure and gene content of *P. copri* remain to be established in order to elucidate the biological functions encoded by its genome that can account for its capacity to ferment dietary polysaccharides [49], its role in inflammation [50, 51] and its impact on host metabolism.

## Electronic supplementary material

ESM (PDF 3.15 mb)

## Data Availability

All data generated or analysed during this study are included in this published article and its supplementary information files.

## Abbreviations

CYP7A1: Cholesterol 7α-hydroxylase
FXR: Farnesoid X receptor
GK: Goto–Kakizaki
VSG: Vertical sleeve gastrectomy

## References

[1] Sonnenburg JL, Bäckhed F (2016). Diet–microbiota interactions as moderators of human metabolism. Nature.

[2] Zmora N, Bashiardes S, Levy M, Elinav E (2017). The role of the immune system in metabolic health and disease. Cell Metab.

[3] Wu H, Tremaroli V, Bäckhed F (2015). Linking microbiota to human diseases: a systems biology perspective. Trends Endocrinol Metab.

[4] Hansen TH, Gøbel RJ, Hansen T, Pedersen O (2015). The gut microbiome in cardio-metabolic health. Genome Med.

[5] Brunkwall L, Orho-Melander M (2017). The gut microbiome as a target for prevention and treatment of hyperglycaemia in type 2 diabetes: from current human evidence to future possibilities. Diabetologia.

[6] Pollak M (2017). The effects of metformin on gut microbiota and the immune system as research frontiers. Diabetologia.

[7] Mulla CM, Middelbeek RJW, Patti ME (2017). Mechanisms of weight loss and improved metabolism following bariatric surgery. Ann N Y Acad Sci.

[8] Nguyen NT, Varela JE (2017). Bariatric surgery for obesity and metabolic disorders: state of the art. Nat Rev Gastroenterol Hepatol.

[9] Schauer PR, Bhatt DL, Kirwan JP (2017). Bariatric surgery versus intensive medical therapy for diabetes—5-year outcomes. N Engl J Med.

[10] Goto Y, Kakizaki M, Masaki N (1976). Production of spontaneous diabetic rats by repetition of selective breeding. Tohoku J Exp Med.

[11] Bihoreau MT, Dumas ME, Lathrop M, Gauguier D (2017). Genomic regulation of type 2 diabetes endophenotypes: contribution from genetic studies in the Goto–Kakizaki rat. Biochimie.

[12] Calderari S, Ria M, Gérard C (2017). Molecular genetics of the transcription factor GLIS3 identifies its dual function in beta cells and neurons. Genomics.

[13] Trung VN, Yamamoto H, Yamaguchi T (2013). Effect of sleeve gastrectomy on body weight, food intake, glucose tolerance, and metabolic hormone level in two different rat models: Goto–Kakizaki and diet-induced obese rat. J Surg Res.

[14] Saeidi N, Meoli L, Nestoridi E (2013). Reprogramming of intestinal glucose metabolism and glycemic control in rats after gastric bypass. Science.

[15] Salinari S, le Roux CW, Bertuzzi A, Rubino F, Mingrone G (2014). Duodenal–jejunal bypass and jejunectomy improve insulin sensitivity in Goto–Kakizaki diabetic rats without changes in incretins or insulin secretion. Diabetes.

[16] Manichanh C, Reeder J, Gibert P (2010). Reshaping the gut microbiome with bacterial transplantation and antibiotic intake. Genome Res.

[17] Bartolí R, Boix J, Odena G, de Vega MV, Lorenzo-Zúñiga V (2012). Determination of the ideal preparation for colonoscopy in a rat model. Surg Laparosc Endosc Percutan Tech.

[18] Brial F, Le Lay A, Hedjazi L (2019). Systems genetics of hepatic metabolome reveals octopamine as a target for non-alcoholic fatty liver disease treatment. Sci Rep.

[19] Sarafian MH, Lewis MR, Pechlivanis A (2015). Bile acid profiling and quantification in biofluids using ultra-performance liquid chromatography tandem mass spectrometry. Anal Chem.

[20] Benjamini Y, Hochberg Y (1995). Controlling the false discovery rate: a practical and powerful approach to multiple testing. J R Stat Soc.

[21] Pedersen HK, Gudmundsdottir V, Nielsen HB (2016). Human gut microbes impact host serum metabolome and insulin sensitivity. Nature.

[22] Kovatcheva-Datchary P, Nilsson A, Akrami R (2015). Dietary fiber-induced improvement in glucose metabolism is associated with increased abundance of Prevotella. Cell Metab.

[23] Rautio M, Lönnroth M, Saxén H, Nikku R, Väisänen M–L (1997). Characteristics of an unusual anaerobic pigmented gram-negative rod isolated from normal and inflamed appendices. Clin Infect Dis.

[24] Bozadjieva N, Heppner KM, Seeley RJ (2018). Targeting FXR and FGF19 to treat metabolic diseases—lessons learned from bariatric surgery. Diabetes.

[25] Chiang JY (2013). Bile acid metabolism and signaling. Compr Physiol.

[26] Teodoro JS, Rolo AP, Palmeira CM (2011). Hepatic FXR: key regulator of whole-body energy metabolism. Trends Endocrinol Metab.

[27] Fang S, Suh JM, Reilly SM (2015). Intestinal FXR agonism promotes adipose tissue browning and reduces obesity and insulin resistance. Nat Med.

[28] Labrecque J, Laforest S, Michaud A, Biertho L, Tchernof A (2017). Impact of bariatric surgery on white adipose tissue inflammation. Can J Diabetes.

[29] Hallberg SJ, Gershuni VM, Hazbun TL, Athinarayanan SJ (2019) Reversing type 2 diabetes: a narrative review of the evidence. Nutrients 11(4). 10.3390/nu11040766 10.3390/nu11040766 PMC6520897 30939855

[30] Le Chatelier E, Nielsen T, Qin J (2013). Richness of human gut microbiome correlates with metabolic markers. Nature.

[31] Pallister T, Jackson MA, Martin TC (2017). Hippurate as a metabolomic marker of gut microbiome diversity: modulation by diet and relationship to metabolic syndrome. Sci Rep.

[32] Tremaroli V, Karlsson F, Werling M (2015). Roux-en-Y gastric bypass and vertical banded gastroplasty induce long-term changes on the human gut microbiome contributing to fat mass regulation. Cell Metab.

[33] Liou AP, Paziuk M, Luevano JM, Machineni S, Turnbaugh PJ, Kaplan LM (2013). Conserved shifts in the gut microbiota due to gastric bypass reduce host weight and adiposity. Sci Transl Med.

[34] Osto M, Abegg K, Bueter M, le Roux CW, Cani PD, Lutz TA (2013). Roux-en-Y gastric bypass surgery in rats alters gut microbiota profile along the intestine. Physiol Behav.

[35] Arora T, Seyfried F, Docherty NG (2017). Diabetes-associated microbiota in fa/fa rats is modified by Roux-en-Y gastric bypass. ISME J.

[36] Miyachi T, Nagao M, Shibata C (2016). Biliopancreatic limb plays an important role in metabolic improvement after duodenal–jejunal bypass in a rat model of diabetes. Surgery.

[37] Tett A, Huang KD, Asnicar F (2019). The Prevotella copri complex comprises four distinct clades underrepresented in westernized populations. Cell Host Microbe.

[38] David LA, Maurice CF, Carmody RN (2014). Diet rapidly and reproducibly alters the human gut microbiome. Nature.

[39] Wu GD, Chen J, Hoffmann C (2011). Linking long-term dietary patterns with gut microbial enterotypes. Science.

[40] De Filippo C, Cavalieri D, Di Paola M (2010). Impact of diet in shaping gut microbiota revealed by a comparative study in children from Europe and rural Africa. Proc Natl Acad Sci U S A.

[41] Leite AZ, Rodrigues NC, Gonzaga MI (2017). Detection of increased plasma interleukin-6 levels and prevalence of *Prevotella copri* and *Bacteroides vulgatus* in the feces of type 2 diabetes patients. Front Immunol.

[42] Otto GW, Kaisaki PJ, Brial F (2019). Conserved properties of genetic architecture of renal and fat transcriptomes in rat models of insulin resistance. Dis Model Mech.

[43] Albaugh VL, Banan B, Ajouz H, Abumrad NN, Flynn CR (2017). Bile acids and bariatric surgery. Mol Asp Med.

[44] Jonker JW, Liddle C, Downes M (2012). FXR and PXR: potential therapeutic targets in cholestasis. J Steroid Biochem Mol Biol.

[45] Ryan KK, Tremaroli V, Clemmensen C (2014). FXR is a molecular target for the effects of vertical sleeve gastrectomy. Nature.

[46] Raghow R, Yellaturu C, Deng X, Park EA, Elam MB (2008). SREBPs: the crossroads of physiological and pathological lipid homeostasis. Trends Endocrinol Metab.

[47] Xu X, So JS, Park JG, Lee AH (2013). Transcriptional control of hepatic lipid metabolism by SREBP and ChREBP. Semin Liver Dis.

[48] Ramracheya RD, McCulloch LJ, Clark A (2016). PYY-dependent restoration of impaired insulin and glucagon secretion in type 2 diabetes following Roux-En-Y gastric bypass surgery. Cell Rep.

[49] Dodd D, Mackie RI, Cann IK (2011). Xylan degradation, a metabolic property shared by rumen and human colonic Bacteroidetes. Mol Microbiol.

[50] Scher JU, Sczesnak A, Longman RS (2013). Expansion of intestinal Prevotella copri correlates with enhanced susceptibility to arthritis. Elife.

[51] Wen C, Zheng Z, Shao T (2017). Quantitative metagenomics reveals unique gut microbiome biomarkers in ankylosing spondylitis. Genome Biol.

