# Supplementary material for: Dominant gut Prevotella copri in gastrectomised non-obese diabetic Goto–Kakizaki rats improves glucose homeostasis through enhanced FXR signalling
Source: Diabetologia. 2020 Mar 16;63(6):1223–35. doi: 10.1007/s00125-020-05122-7 (PMC7228998; doi:10.1007/s00125-020-05122-7)
Supplement: Supplementary file 1 — (PDF 3.15 mb) [file 125_2020_5122_MOESM1_ESM.pdf]

ESM Material and Methods

Animals

Goto-Kakizaki (GK/Ox) rats were maintained in individually ventilated cages in a controlled environment (12 h dark-light cycles 22–24°C; humidity 50–60%). They had ad libitum access to water and standard chow (SAFE, Augy, France). Animals from the experimental and control groups were housed in different cages to avoid unintentional microbiota transplants via coprophagy. Animals were randomly assigned to experimental and control groups and all glucose tolerance tests and analytical assays were blinded. Animal procedures were authorized by the University Ethics Committee in Animal Experiment (4231201602231507187).

VSG

Vertical sleeve gastrectomy (VSG) was performed in 16-week-old male GK rats. Rats were anesthetized by isofluorane intoxication and the lateral 80% of the stomach was excised with a linear cutter (TLC55, Ethicon, Issy Les Moulineaux, France) to leave a tubular gastric remnant in continuity with the oesophagus, the pylorus and the duodenum. Post-surgical analgesia was applied over the following three days with enrofloxacine 2.5% (5mg/kg body weight) and buprenorphine (200µg/kg body weight). Some animals were excluded from the protocol following gastrectomy and were culled when excessive loss of body condition was observed. Control GK rats were sham operated using a procedure involving isolation of the stomach followed by application of pressure with blunt forceps along a vertical line between the oesophageal sphincter and the pylorus. Rats were killed by cervical dislocation 13 weeks after the surgical procedures and the content of caecum and colon were rapidly harvested, quick-frozen in liquid nitrogen and stored at -80°C.

Pair-feeding studies

Thirty days after surgery, sham rats were pair-fed to match the food intake of free-fed VSG GK rats. The equivalent amount of food consumed by GK rats of the VSG group was given to sham operated GK rats in a single dose at 6 pm, which corresponds to the beginning of the activity phase of the animals. Blood glucose was determined using an Accu-Check® Performa (Roche Diagnostics, Meylan, France).

Gut microbiota transfer

Five month old male GK rats were treated orally with Inexium (Omeprazole, Astrazeneca, Courbevoie, France) (2mL/rat) for three days followed by gavage with Moviprep (Norgine, Rueil-Malmaison, France) (2mL/rat) [1, 2]. Caecal microbiota (250mg) from VSG treated or sham operated GK rats were homogenised in 1mL of saline and centrifuged. The supernatant of each sample was collected (no pool) and individually used to inoculate both orally (300µL) and by rectal injection (300µL) GK rats under general anesthesia (ketamine/xylazine). Inoculations were carried out one week after the Moviprep treatment.

Enrichment of gut microbiota with *P. copri*

P. copri is tolerant to vancomycin and kanamycin antibiotics [3]. Randomly selected five month old male GK rats were given vancomycin (0.5g/L) and kanamycin (1g/L) [4] in the drinking water for ten days. GK controls remained on antibiotic-free maintenance conditions.

***P. copri* supplementation**

Commercially available *P. copri* (DSM 18205, DSMZ, Braunschweig, Germany) was cultivated under anaerobic conditions on BD Schaedler Kanamycin-Vancomycin Agar with 5% Sheep Blood (Biomerieux, Marcy l'Etoile, France). We adapted to the rat the procedure of *P. copri* supplementation optimized in mice by Kovatcheva-Datchary and colleagues [5]. Five month old male GK rats were given vancomycin (0.5g/l), neomycin (1g/l), metronidazole (1g/l) and ampicillin (1g/l) [6] in the drinking water during ten days. Then,

randomly selected rats were inoculated with a single gavage of 5.10^8 CFU *P. copri*. The control group received an equivalent dosage of heat-killed *P. copri*.

Glucose tolerance tests and sample collection and analysis

Oral and intraperitoneal glucose tolerance tests (OGTT, IPGTT) were performed in conscious rats after an overnight fast. Due to changes in intra-abdominal organs secondary to gastrectomy, including extensive connective tissue, OGTT were preferred to IPGTT to assess glucose tolerance and glucose-induced insulin secretion in VSG treated rats (Experiment 1, Fig. 1). OGTT was also used to assess glucose tolerance in sham operated controls. For OGTT, a bolus of glucose (1g/kg body weight) was given by gavage in conscious rats. Blood samples were collected from the tail vein before glucose administration and 15, 30, 60, 90 and 120 min afterwards. For IPGTT carried out in GK rats inoculated with the caecal content of VSG or sham operated GK (Experiment 2, Fig. 1), treated with *P. copri* permissive antibiotics or no antibiotics (Experiment 3, Fig. 1) and inoculated with *P. copri* or heat inactivated *P. copri* (Experiment 4, Fig. 1), conscious rats were injected intraperitoneally with a solution of glucose (1g/kg body weight) after an overnight fast. Blood samples were collected from the tail vein before glucose injection and 5, 15, 30, 60, 90, 120, 180 and 240 minutes afterwards. Glycemia was determined using an Accu-Check® Performa (Roche Diagnostics, Meylan, France) and insulinemia was assayed by ELISA (Mercodia, Uppsala, Sweden). Glucose tolerance was assessed with the cumulative glycemia (increment of glucose values during the test) and the ΔG (cumulative glycemia above baseline).

Three days after the glucose tolerance tests, rats were fasted overnight and killed by lethal injection of sodium pentobarbital. Plasma samples, adipose tissue, liver and caecal content were quick-frozen in liquid nitrogen and stored at -80°C. Colorimetric assays were used to determine plasma and liver triglycerides (ab65336; Abcam, Paris, France) and liver glycogen (Sigma-Aldrich).

Metagenome sequencing

Bacterial DNA was independently prepared from caecal (10 from VSG-treated GK rats and 8 from sham operated GK controls) and colon (6 from VSG-treated GK rats and 3 from sham operated GK controls) samples (DNAStool mini kit, QIAGEN, Courtaboeuf, France). Whole-genome shotgun sequencing was performed on Illumina HiSeq 2000 instruments (Illumina, San Diego, CA) to generate 150nt sequence reads. For each sample, a single sequencing library was produced and bar-coded. Pools of four samples were run per lane. Every sample was sequenced on multiple instruments to minimize potential technical flow cell or instrument biases. Data was de-duplicated using standard in-house protocols. There were four separate sets of cluster building per sample in order to reduce errors of duplication and de-duplication. To estimate the biodiversity of the samples, the WGS data was mined for sequence motifs corresponding to a 55nt segment of the bacterial 16S rRNA variable 1-3 region. A total of 8,767 unique rDNA motifs were found (517,697 occurrences), the vast majority of which were rare, both in terms of number of samples detected and prevalence in individual samples. To reduce data sparsity, we analyzed motifs that accounted for a minimum prevalence of 0.5% or better in any individual sample. For preliminary indications of species identifications, motifs were aligned to publicly available microbial databases to retrieve perfect and highly similar matches.

Quantitative PCR

Bacterial DNA from fresh caecum samples were extracted using DNA Stool mini kit (QIAGEN, Courtaboeuf, France). DNA yield and integrity were determined using a Nanodrop 2000c (Thermo Fisher Scientific, Illkirch, France) and agarose gel electrophoresis. *P. copri* enrichment was assessed by quantitative PCR using SYBR green assays (Life technologies, Saint Aubin, France) and comparison to universal 16S rRNA. Preparation of liver and adipose tissue RNA and quantitative RT-PCR analyses were performed as described

[7] using the housekeeping gene HPRT. Relative quantification of mRNA levels of candidate genes were carried out using the Livak and Schmittgen method [8]. Reactions were run on taqman 7900 HT system for *P. copri* analysis and on a CFX96 Real-Time system (Bio-Rad) for gene expression. Oligonucleotide sequences are given in **Supplementary Table 1**.

Quantitative analysis of plasma bile acids

Plasma bile acids were quantified on 100µL plasma aliquots as described [9]. Plasma were spiked with isotopically labelled bile acid standards and treated with ice cold methanol in order to remove proteins. Bile acids were separated in an ACQUITY BEH C8 column and detected by a Xevo TQ-S mass spectrometer (Waters, Manchester, UK) operating in negative ionization mode and multiple reaction monitoring. The method allowed us to quantify up to 35 distinct bile acids.

Statistical analyses

R packages were used to assess differences in the frequency of 16SrDNA motifs between gastrectomised and sham operated control rats and to compute a p-value for each motif with a threshold of significance set to 0.05. P-values were corrected for multiple testing using the Benjamini-Hochberg method [10]. Blood glucose and insulin secretion data during the glucose tolerance tests were analysed with the Kruskal-Wallis test in order to account for conserved intra-individual distribution of blood glucose and plasma insulin. Non-parametric Mann-Whitney U tests were used to assess differences in physiological phenotypes, bile acid data and gene expression between experimental and control groups.

References

[1] Manichanh C, Reeder J, Gibert P, et al. (2010) Reshaping the gut microbiome with bacterial transplantation and antibiotic intake. Genome Res 20(10): 1411-1419. 10.1101/gr.107987.110

[2] Bartolí R, Boix J, Odena G, de Vega Moreno V, Lorenzo-Zúñiga V (2012) Determination of the ideal preparation for colonoscopy in a rat model. Surg Laparosc Endosc Percutan Tech 22(6): 542-545. 10.1097/SLE.0b013e318264c4f9

[3] Tan H, Zhao J, Zhang H, Zhai Q, Chen W (2018) Isolation of Low-Abundant Bacteroidales in the Human Intestine and the Analysis of Their Differential Utilization Based on Plant-Derived Polysaccharides. Front Microbiol 9: 1319. 10.3389/fmicb.2018.01319
[4] Rautio M, Lönnroth M, Saxén H, et al. (1997) Characteristics of an unusual anaerobic pigmented gram-negative rod isolated from normal and inflamed appendices. Clin Infect Dis 25 Suppl 2: S107-110
[5] Kovatcheva-Datchary P, Nilsson A, Akrami R, et al. (2015) Dietary Fiber-Induced Improvement in Glucose Metabolism Is Associated with Increased Abundance of Prevotella. Cell Metab 22(6): 971-982. 10.1016/j.cmet.2015.10.001
[6] Rakoff-Nahoum S, Paglino J, Eslami-Varzaneh F, Edberg S, Medzhitov R (2004) Recognition of commensal microflora by toll-like receptors is required for intestinal homeostasis. Cell 118(2): 229-241. 10.1016/j.cell.2004.07.002
[7] Brial F, Le Lay A, Hedjazi L, et al. (2019) Systems Genetics of Hepatic Metabolome Reveals Octopamine as a Target for Non-Alcoholic Fatty Liver Disease Treatment. Sci Rep 9(1): 3656. 10.1038/s41598-019-40153-0
[8] Livak KJ, Schmittgen TD (2001) Analysis of relative gene expression data using real-time quantitative PCR and the 2(-Delta Delta C(T)) Method. Methods 25(4): 402-408. 10.1006/meth.2001.1262
[9] Sarafian MH, Lewis MR, Pechlivanis A, et al. (2015) Bile acid profiling and quantification in biofluids using ultra-performance liquid chromatography tandem mass spectrometry. Anal Chem 87(19): 9662-9670. 10.1021/acs.analchem.5b01556
[10] Benjamini Y, Hochberg Y (1995) Controlling the false discovery rate: a practical and powerful approach to multiple testing. J R Stat Soc 57(1): 289-300

ESM Table 1. Oligonucleotides used for quantitative RT PCR.

	**Forward**	**Reverse**
Fxr	TACCATTACAACGCGCTCAC	GCCCCCGTTCTTACACTTG
Shp	GCAGCACTGCCTGGAGTC	GTGTGCAATGTGGCAGGA
Pepck	GAAGGAGTGCCCATCGAA	TCCTACAAACACCCCATGCT
G6pc	TCAAGAGACTGTGGGCATCA	GGCGCTGTCCAAAAAGAAT
Srebf1	CCGTTTCTTCGTGGATGG	CACAGAATAGTCGGGTCACCT
chrebp	ACTGTTCCCTGCCTGCTCT	CCTTGTGGCTTGCTCAGG
Sorbs1	CTTGCAGCACAACCGAGAT	GATGGGCGAGGTGAGTTCT
Pygl	GAGTGCTGTACCCCAACGAT	CCTTGAAACGTCGGATGAC
Hprt	GGTCCATTCCTATGACTGTAGATTTT	AACAATCAAGACGTTCTTTCCAG
16S	ACTCCTACGGGAGGCAGCAGT	ATTACCGCGGCTGCTGGC
P.Copri	CCGGACTCCTGCCCCTGCAA	GTTGCGCCAGGCACTGCGAT

ESM Table 2. Frequency of rDNA motifs in gut microbiota from gastrectomised (Vertical sleeve gastrectomy, VSG) and sham operated rats of the Goto-Kakizaki strain. The rDNA motifs shown are present at a frequency >0.005% in at least 50% of individual rats. Frequencies of rDNA motifs from caecum samples were used to calculate means and SEM.

		SHAM		VSG	
rDNA Motif	rDNA motif Sequence	Mean	SEM	Mean	SEM
V13A7759	ATTACCGCGGCTGCTGGCACGGAATTAGCCGGTCCTTATTCATAAGGTACATGCA	9.073960%	3.113758%	22.705789%	5.538537%
V13A2810	ATTACCGCGGCTGCTGGCACGTAGTTAGCCGGGGCTTCTTACTCAGGTACCGTCA	7.492013%	1.036242%	6.994838%	1.253067%
V13A5897	ATTACCGCGGCTGCTGGCACGTAGTTAGCCGGGGCTTCTTGGTCAGGTACCGTCA	7.062306%	1.079612%	5.177857%	1.333343%
V13A12285	ATTACCGCGGCTGCTGGCACGTAGTTAGCCGGGGCTTCTTAGTCAGGTACCGTCA	5.790701%	0.854445%	5.759024%	1.063598%
V13A395	ATTACCGCGGCTGCTGGCACGGAGTTAGCCGATGCTTTTTCTCCGGGTACTCTCC	3.075614%	1.097742%	3.565136%	0.726443%
V13A6709	ATTACCGCGGCTGCTGGCACGGAATTAGCCGGTCCTTATTCATACGGTACCTGCA	3.042629%	0.537824%	1.493961%	0.236652%
V13A14448	ATTACCGCGGCTGCTGGCACGGAATTAGCCGGTCCTTATTCATGCGGTACCTGCA	2.135061%	0.558220%	2.724241%	0.436746%
V13A2287	ATTACCGCGGCTGCTGGCACGGAGTTAGCCGATGCTTATTCATACGGTACATACA	2.858376%	0.571613%	2.147298%	0.404692%
V13A14726	ATTACCGCGGCTGCTGGCACGGAGTTAGCCGATGCTTTTTCTCAGGGTACTCTCC	3.007887%	0.701885%	2.377838%	0.588273%
V13A7919	ATTACCGCGGCTGCTGGCACGGAGTTAGCCGATGCTTTTTCTTCAGGTACACTCA	2.128682%	0.688518%	4.629847%	1.245758%
V13A5402	ATTACCGCGGCTGCTGGCACGGAGTTAGCCGATGCTTTTTCTTCGGGTACTCTCG	1.868137%	0.344738%	1.920562%	0.280246%
V13A1689	ATTACCGCGGCTGCTGGCACGGAGTTAGCCGATGCTTTTTCTTCAGGTACTCTCG	1.161420%	0.751805%	1.255658%	0.345201%
V13A7641	ATTACCGCGGCTGCTGGCACGTAGTTAGCCGTGACTTTCTGGTTGATTACCGTCA	0.797960%	0.074973%	1.448365%	0.803381%
V13A4836	ATTACCGCGGCTGCTGGCACGGAGTTAGCCGATCCTTATTCGTATGGTACATACA	0.520971%	0.514560%	1.641454%	0.550971%
V13A4809	ATTACCGCGGCTGCTGGCACGTAGTTAGCCGGGGCTTCTTAATCAGGTACCGTCT	1.771341%	0.940886%	1.165276%	0.448003%
V13A7817	ATTACCGCGGCTGCTGGCACGGAGTTAGCCGATGCTTTTTCTCCGGGTACACTCA	0.598281%	0.091503%	0.557536%	0.240674%
V13A8451	ATTACCGCGGCTGCTGGCACGTAGTTAGCCGTGGCTTAACCTCAGGTACCGTCAT	1.814001%	0.614578%	0.096369%	0.039797%
V13A9035	ATTACCGCGGCTGCTGGCACGTAGTTAGCCGTTGCTTTTTCTCCAGGTACCGTCA	0.757123%	0.343471%	0.396081%	0.100330%
V13A9309	ATTACCGCGGCTGCTGGCACGGAGTTAGCCGATGCTTTTTCTTCGGGTACACGCA	0.349578%	0.243147%	1.017451%	0.228088%
V13A3764	ATTACCGCGGCTGCTGGCACGGAGTTAGCCGATGCTTTTTCTCCGGGTACCCTCA	0.844584%	0.298465%	0.485913%	0.133419%
V13A2469	ATTACCGCGGCTGCTGGCACGGAATTAGCCGATGCTTATTCATAAGGTACATACA	1.514555%	0.612323%	0.460579%	0.247414%
V13A8741	ATTACCGCGGCTGCTGGCACGGAGTTAGCCGATGCTTTTTCTTCGGGTACACTCA	0.652240%	0.252331%	0.402395%	0.257189%
V13A15371	ATTACCGCGGCTGCTGGCACGTAGTTAGCCGTGGCTTATTCAAGAGGTACCGTCT	0.962511%	0.167741%	0.681418%	0.132381%
V13A3935	ATTACCGCGGCTGCTGGCACGGAGTTAGCCGATCCTTATTCATACGGTACATACA	0.888069%	0.212414%	0.406619%	0.120174%

V13A5381	ATTACCGCGGCTGCTGGCACGGAGTTAGCCGATGCTTTTTCTTCGGGTACTCTCA	1.277505%	0.323793%	0.589433%	0.165869%
V13A399	ATTACCGCGGCTGCTGGCACGGAGTTAGCCGATGCTTTTTCTCCGGGTACTCTCG	0.901794%	0.117232%	0.483733%	0.167078%
V13A10361	ATTACCGCGGCTGCTGGCACGAAGTTAGCCGGTGCTTTTTCTGTCGCTACTGTCA	0.658245%	0.137179%	0.326717%	0.120989%
V13A1694	ATTACCGCGGCTGCTGGCACGGAGTTAGCCGATGCTTTTTCTTCAGGTACTCTCA	0.726491%	0.378283%	0.159166%	0.094443%
V13A296	ATTACCGCGGCTGCTGGCACGTAGTTAGCCGGTGCTTATTCTTAGAGTACCGTCA	0.471294%	0.034958%	0.613924%	0.158336%
V13A13773	ATTACCGCGGCTGCTGGCACGTAGTTAGCCGTGGCTTTCTGGTTAGATACCGTCG	0.537240%	0.180613%	0.480570%	0.142488%
V13A12346	ATTACCGCGGCTGCTGGCACGTAGTTAGCCGTGGCTTATTCAAGGGGTACCGTCT	0.612998%	0.079235%	0.868195%	0.199052%
V13A13515	ATTACCGCGGCTGCTGGCACGTAGTTAGCCGTGGCTTATTCCTCAGGTACCGTCA	0.662303%	0.122855%	0.769364%	0.127965%
V13A2602	ATTACCGCGGCTGCTGGCACGTAGTTAGCCGGAGCTTTCTTGAAAGGTACCGTCA	0.580478%	0.184266%	0.459190%	0.127540%
V13A7275	ATTACCGCGGCTGCTGGCACGGAGTTAGCCGATCCTTATTCATAAAGTACATGCA	0.685053%	0.126167%	0.328284%	0.093346%
V13A3762	ATTACCGCGGCTGCTGGCACGGAGTTAGCCGATGCTTTTTCTCCGGGTACCCTCG	0.641765%	0.260571%	0.299114%	0.120361%
V13A15465	ATTACCGCGGCTGCTGGCACGGAGTTAGCCGGTGCTTCCTCTGTGGGTACCGTCA	0.467655%	0.098343%	0.422795%	0.113124%
V13A13991	ATTACCGCGGCTGCTGGCACGTAGTTAGCCGGGGCTTCTTGGTCAGGTACCGTCC	1.122187%	1.044288%	0.091681%	0.034861%
V13A14005	ATTACCGCGGCTGCTGGCACGTAGTTAGCCGGGGCTTCTTGGTCAGGTACCGTCT	0.921962%	0.489481%	0.417073%	0.253385%
V13A6464	ATTACCGCGGCTGCTGGCACCAGACTTGCCCTCCAATGGATCCTCGTTAAAGGAT	0.363374%	0.222759%	0.368286%	0.136257%
V13A8908	ATTACCGCGGCTGCTGGCACGTAGTTAGCCGTGACTTTCTGGTTGGATACCGTCA	0.423517%	0.132131%	0.373155%	0.074619%
V13A3428	ATTACCGCGGCTGCTGGCACGGAGTTAGCCGATGCTTTTTCTTCGGGTACTCTCC	0.427074%	0.125405%	0.659545%	0.255987%
V13A9942	ATTACCGCGGCTGCTGGCACGGAGTTAGCCGATGCTTTTTCTTCAGGTACCCTCA	0.322128%	0.207905%	0.232828%	0.118087%
V13A2378	ATTACCGCGGCTGCTGGCACGTATTTAGCCGGTGCTTCTTAGTCAGGTACCGTCA	0.319487%	0.118423%	0.433062%	0.148852%
V13A12274	ATTACCGCGGCTGCTGGCACGTAGTTAGCCGGGGCTTCTTAGTCAGGTACCGTCT	0.398442%	0.070005%	0.571416%	0.135201%
V13A4785	ATTACCGCGGCTGCTGGCACGTAGTTAGCCGTGGCTTATTCGTCAGGTACCGTCA	0.499526%	0.067431%	0.374966%	0.050785%
V13A12862	ATTACCGCGGCTGCTGGCACGTATTTAGCCGGAGCTTCTTAGTCAGGTACCGTCA	0.492408%	0.151763%	0.232185%	0.070782%
V13A13172	ATTACCGCGGCTGCTGGCACGTAGTTAGCCGGGGCTGCTTACTCAGGTACCGTCA	0.705369%	0.288026%	0.370483%	0.190582%
V13A13919	ATTACCGCGGCTGCTGGCACGGAGTTAGCCGATCCTTATTCGTACGATACTTTCA	0.431286%	0.146402%	0.207771%	0.037019%
V13A14410	ATTACCGCGGCTGCTGGCACGGAGTTAGCCGATGCTTATTCTCCGGGTACTCTCA	0.003467%	0.001814%	0.636456%	0.547968%
V13A4765	ATTACCGCGGCTGCTGGCACGTAGTTAGCCGTGGCTTCCTCCGCGGGTACCGTCA	0.258695%	0.107711%	0.355995%	0.115338%
V13A4931	ATTACCGCGGCTGCTGGCACGGAGTTAGCCGGTGCTTCTTCTGCGGGTAACGTCA	0.162788%	0.125621%	0.532038%	0.453717%
V13A9079	ATTACCGCGGCTGCTGGCACGTAGTTAGCCGTGGCTTCCTAAAGGGGTACCGTCA	0.447368%	0.199142%	0.284447%	0.059115%
V13A4794	ATTACCGCGGCTGCTGGCACGTAGTTAGCCGTGGCTTATTCGTCAGGTACCGTCT	0.318805%	0.058714%	0.477093%	0.114728%
V13A7810	ATTACCGCGGCTGCTGGCACGTAGTTAGCCGTGGCTTATTCAACAGGTACCGTCA	0.389182%	0.097889%	0.292454%	0.033631%

V13A397	ATTACCGCGGCTGCTGGCACGGAGTTAGCCGATGCTTTTTCTCCGGGTACTCTCA	0.231137%	0.047982%	0.353059%	0.193770%
V13A5701	ATTACCGCGGCTGCTGGCACGTAGTTAGCCGGGGCTTCTTAGTCAGGTACTGTCA	0.436434%	0.224545%	0.388864%	0.244682%
V13A8825	ATTACCGCGGCTGCTGGCACGTAGTTAGCCGTGGCTTATTCATCAGGTACCGTCA	0.365925%	0.045631%	0.278714%	0.051819%
V13A3265	ATTACCGCGGCTGCTGGCACGTAGTTAGCCGAGGCTTATTCCTAAGGTACCGTCA	0.372160%	0.099899%	0.352172%	0.111873%
V13A7921	ATTACCGCGGCTGCTGGCACGGAGTTAGCCGATGCTTTTTCTTCAGGTACACTCG	0.522578%	0.215917%	0.152104%	0.060037%
V13A14225	ATTACCGCGGCTGCTGGCACGGAGTTAGCCGATGCTTTTTCTTCGGGTACTCGCA	0.064088%	0.046996%	0.310420%	0.096528%
V13A3045	ATTACCGCGGCTGCTGGCACGTAGTTAGCCGTGGCTTATTCATTCGGTACCGTCA	0.375331%	0.049509%	0.239554%	0.053794%
V13A9455	ATTACCGCGGCTGCTGGCACGTAGTTAGCCGTGGCTTATTCCTAAGGTACCGTCA	0.461836%	0.183591%	0.313700%	0.135863%
V13A9476	ATTACCGCGGCTGCTGGCACGTAGTTAGCCGTGGCTTTCTTGAATAGTACCATCA	0.274998%	0.097695%	0.183092%	0.117623%
V13A4570	ATTACCGCGGCTGCTGGCACGTATTTAGCCGGGGCTTCTTAGTCAAGTACCGTCA	0.370027%	0.304566%	0.045416%	0.028404%
V13A10016	ATTACCGCGGCTGCTGGCACGGAGTTAGCCGATCCTTATTCATATGGTACATACA	0.251930%	0.065103%	0.122937%	0.020297%
V13A9779	ATTACCGCGGCTGCTGGCACGTAGTTAGCCGTGGCTTTCTTGAATGGTACCGTCA	0.238195%	0.088096%	0.159937%	0.077970%
V13A15364	ATTACCGCGGCTGCTGGCACGTAGTTAGCCGTGGCTTATTCAAGAGGTACCGTCA	0.326098%	0.100808%	0.198171%	0.041510%
V13A5861	ATTACCGCGGCTGCTGGCACGTAGTTAGCCGGAGCTTCCTCCTCTGCTACCGTCA	0.240907%	0.090468%	0.222516%	0.049927%
V13A2959	ATTACCGCGGCTGCTGGCACGTAGTTAGCCGTGGCTTCCTTAAGGGGTACCGTCA	0.368274%	0.119296%	0.161128%	0.089590%
V13A4574	ATTACCGCGGCTGCTGGCACGTAGTTAGCCGGAGCTTCCTCCTCGGGTACCGTCA	0.140794%	0.088784%	0.418000%	0.256027%
V13A890	ATTACCGCGGCTGCTGGCACGTAGTTAGCCGGAGCTTATTCTACAGGTACTGTCT	0.186033%	0.046808%	0.161842%	0.033215%
V13A1692	ATTACCGCGGCTGCTGGCACGGAGTTAGCCGATGCTTTTTCTTCAGGTACTCTCC	0.318164%	0.102340%	0.122003%	0.035755%
V13A11018	ATTACCGCGGCTGCTGGCACGTAGTTAGCCGTGACTTTCTAAGTAATTACCGTCA	0.054835%	0.021761%	0.020063%	0.005370%
V13A6953	ATTACCGCGGCTGCTGGCACGTAGTTAGCCGTGGCTTATTCCTGAGGTACCGTCA	0.287954%	0.087473%	0.167002%	0.028440%
V13A12840	ATTACCGCGGCTGCTGGCACGGAGTTAGCCGATGCTTTTTCTCCAGGTACACTCG	0.363406%	0.186690%	0.078483%	0.040605%
V13A12857	ATTACCGCGGCTGCTGGCACGTATTTAGCCGGAGCTTCTTAGTCAGGTACCGTCT	0.285795%	0.140626%	0.022901%	0.006473%
V13A10048	ATTACCGCGGCTGCTGGCACGTAGTTAGCCGGAGCTTATTCATTGGTTACCGTCA	0.664762%	0.265586%	0.004005%	0.003247%
V13A12206	ATTACCGCGGCTGCTGGCACGTAGTTAGCCGATGCTTATTCCTTAGCTACCGTCA	0.141923%	0.116299%	0.205885%	0.052833%
V13A7023	ATTACCGCGGCTGCTGGCACGTAGTTAGCCGGAGCTTCCTCCTTAGGTACTGTCT	0.211395%	0.176694%	0.036946%	0.010320%
V13A4817	ATTACCGCGGCTGCTGGCACGTAGTTAGCCGGGGCTTCTTAATCAGGTACCGTCA	0.327726%	0.114804%	0.180825%	0.093872%
V13A2090	ATTACCGCGGCTGCTGGCACGTAGTTAGCCGTGGCTTTTTCTCCAGGTACCGTCA	0.156921%	0.058349%	0.153020%	0.023402%
V13A9744	ATTACCGCGGCTGCTGGCACGTAGTTAGCCGGAGCTTCCTCCCCAGGTACCGTCA	0.204856%	0.043307%	0.188786%	0.037579%
V13A6778	ATTACCGCGGCTGCTGGCACGTAGTTAGCCGTGGCTTATTCATGAGGTACCGTCA	0.158744%	0.029032%	0.110196%	0.016649%
V13A3763	ATTACCGCGGCTGCTGGCACGGAGTTAGCCGATCCTTATTCATAAGGTACATACA	0.152878%	0.051287%	0.116417%	0.022258%

V13A11183	ATTACCGCGGCTGCTGGCACGTAGTTAGCCGGGGCTTCCTCCTCAAGTACCGTCA	0.362917%	0.288898%	0.041124%	0.015397%
V13A2248	ATTACCGCGGCTGCTGGCACGTAGTTAGCCGTGGCTTATTCCTTGGGTACCGTCA	0.178280%	0.060737%	0.127916%	0.062748%
V13A2804	ATTACCGCGGCTGCTGGCACGTAGTTAGCCGGGGCTTCTTACTCAGGTACCGTCT	0.191374%	0.150683%	0.247214%	0.143975%
V13A2715	ATTACCGCGGCTGCTGGCACGGAGTTAGCCGATGCTTTTTCTCCGGGTACGCTCC	0.192335%	0.086924%	0.040751%	0.006881%
V13A9336	ATTACCGCGGCTGCTGGCACGGAGTTAGCCGGTGCTTCCTCTGCAGGTACAGTCA	0.122746%	0.044292%	0.121646%	0.049237%
V13A12283	ATTACCGCGGCTGCTGGCACGTAGTTAGCCGGGGCTTCTTAGTCAGGTACCGTCC	0.149669%	0.058312%	0.135433%	0.039204%
V13A10886	ATTACCGCGGCTGCTGGCACGGAGTTAGCCGATGCTTCCTCTGTGGGTACCGTCA	0.204921%	0.126672%	0.085847%	0.044966%
V13A3640	ATTACCGCGGCTGCTGGCACGTAGTTAGCCGTGGCTTATTCCTGGGGTACCGTCA	0.254593%	0.157222%	0.147631%	0.093794%
V13A13697	ATTACCGCGGCTGCTGGCACGTAGTTAGCCGGGGCTTCTTACTCAGGTACTGTCA	0.144281%	0.059503%	0.133587%	0.062692%
V13A9310	ATTACCGCGGCTGCTGGCACGGAGTTAGCCGATGCTTTTTCTTCGGGTACGCTCG	0.087376%	0.025648%	0.119551%	0.031588%
V13A234	ATTACCGCGGCTGCTGGCACGGAGTTAGCCGATCCTTATTCATAAAGTACATACA	0.148108%	0.031337%	0.071154%	0.057642%
V13A6810	ATTACCGCGGCTGCTGGCACGTAGTTAGCCGAGGCTTATTCTGCGGGTACAGTCA	0.089681%	0.027008%	0.155656%	0.032834%
V13A9699	ATTACCGCGGCTGCTGGCACGTAGTTAGCCGTGGCTTATTCTTGGGGTACCGTCA	0.151717%	0.065056%	0.106825%	0.036717%
V13A7528	ATTACCGCGGCTGCTGGCACGTAGTTAGCCGGAGCTTATTCTATAGGTACTGTCT	0.063967%	0.024797%	0.025375%	0.014026%
V13A762	ATTACCGCGGCTGCTGGCACGTAGTTAGCCGGAGCTTATTCATTGGGTACCGTCA	0.298162%	0.238334%	0.070131%	0.045810%
V13A9832	ATTACCGCGGCTGCTGGCACGTAGTTAGCCGTGGCTTCCTCCTTGGTTACCGTCA	0.273462%	0.097341%	0.000000%	0.000000%
V13A12281	ATTACCGCGGCTGCTGGCACGTAGTTAGCCGGGGCTTCTTAGTCAGGTACCGTCG	0.120772%	0.061095%	0.210869%	0.134322%
V13A5211	ATTACCGCGGCTGCTGGCACGTAGTTAGCCGTGGCTTATTCCTCGGGTACCGTCA	0.051343%	0.024606%	0.258342%	0.142205%
V13A14912	ATTACCGCGGCTGCTGGCACGTATTTAGCCGGTGCTTCTTCTTCAGGTACCGTCA	0.153996%	0.118104%	0.067371%	0.049555%
V13A8693	ATTACCGCGGCTGCTGGCACGGAGTTAGCCGGTGCTTCCTTTGAAGGTACCGTCA	0.087164%	0.018621%	0.108535%	0.027285%
V13A9224	ATTACCGCGGCTGCTGGCACGTAGTTAGCCGTGGCTTTCTCATTGGGTACAGTCA	0.143025%	0.050414%	0.053836%	0.022704%
V13A7033	ATTACCGCGGCTGCTGGCACGTAGTTAGCCGGAGCTTCCTCCTTAGGTACTGTCC	0.130017%	0.019237%	0.064986%	0.008975%
V13A6692	ATTACCGCGGCTGCTGGCACGTAGTTAGCCGGAGCTTTCTAAATAGGTACCGTCA	0.099832%	0.029342%	0.168992%	0.031914%
V13A2034	ATTACCGCGGCTGCTGGCACGTAGTTAGCCGGAGCTTATTCATTAAGTACCGTCA	0.118148%	0.060067%	0.026863%	0.015383%
V13A3780	ATTACCGCGGCTGCTGGCACGTAGTTAGCCGGGGCTTCTTATTCAGGTACCGTCA	0.331581%	0.302038%	0.020284%	0.004292%
V13A795	ATTACCGCGGCTGCTGGCACGTAGTTAGCCGGGGCTTCTTATTCAGGTACTGTCA	0.119465%	0.070474%	0.161858%	0.075060%
V13A12353	ATTACCGCGGCTGCTGGCACGTAGTTAGCCGTGGCTTATTCCTGGAGTACCGTCA	0.150392%	0.067379%	0.060485%	0.024290%
V13A6268	ATTACCGCGGCTGCTGGCACGGAATTAGCCGGCCCTTATTCGCACGGTACCTGCA	0.230506%	0.105329%	0.077303%	0.041677%
V13A1861	ATTACCGCGGCTGCTGGCACGGAGTTAGCCGGTGCTTCTTCTGTGATTAACGTCA	0.030512%	0.007869%	0.124497%	0.024124%
V13A12247	ATTACCGCGGCTGCTGGCACGTAGTTAGCCGAGGCTTATTCTGTGGGTACCGTCA	0.085460%	0.049299%	0.046031%	0.030991%

V13A686	ATTACCGCGGCTGCTGGCACGTAGTTAGCCGTTGCTTTTTCTGCAGGTACCGTCA	0.136968%	0.052156%	0.021882%	0.010981%
V13A13238	ATTACCGCGGCTGCTGGCACATAGTTAGCCGGGGCTTCCTCCTTAGGTACAGTCT	0.076138%	0.027324%	0.061346%	0.019143%
V13A9407	ATTACCGCGGCTGCTGGCACGGAATTAGCCGATGCTTATTCTTCGGATACTTGCA	0.088140%	0.042325%	0.100783%	0.031700%
V13A4788	ATTACCGCGGCTGCTGGCACGTAGTTAGCCGTGGCTTGTTTTCAGGGTACCGTCT	0.118252%	0.028708%	0.074330%	0.020813%
V13A12329	ATTACCGCGGCTGCTGGCACGTAGTTAGCCGTGGCTTATTCAAGGGGTACCGTCA	0.041330%	0.024689%	0.191548%	0.152264%
V13A10205	ATTACCGCGGCTGCTGGCACGTAGTTAGCCGGAGCTTATTCTTCGGGTACCGTCA	0.090057%	0.028146%	0.079705%	0.029520%
V13A5823	ATTACCGCGGCTGCTGGCACATAGTTAGCCGGAGCTTCCTCCTGAGGTACTGTCA	0.082170%	0.034722%	0.037340%	0.013421%
V13A6595	ATTACCGCGGCTGCTGGCACGTAGTTAGCCGGAGCTTTTTCTCCAGGTACCGTCA	0.105116%	0.029292%	0.051328%	0.009318%
V13A9082	ATTACCGCGGCTGCTGGCACGTAGTTAGCCGAGGCTTATTCTGTGGGTACAGTCA	0.112193%	0.057894%	0.031658%	0.015528%
V13A7077	ATTACCGCGGCTGCTGGCACGTAGTTAGCCGGGGCTTCTTGTTCAGGTACCGTCA	0.004856%	0.002685%	0.149103%	0.146447%
V13A13337	ATTACCGCGGCTGCTGGCACGGAGTTAGCCGATGCTTATTCGTCAAGTACTGGCA	0.042316%	0.010632%	0.062282%	0.012847%
V13A11382	ATTACCGCGGCTGCTGGCACGTATTTAGCCGGAGCTTCTTATTCAGGTACTGTCA	0.009740%	0.009740%	0.087731%	0.038982%
V13A12760	ATTACCGCGGCTGCTGGCACGTAGTTAGCCGGGGCTTCTTTTTCAGGTACCTTCT	0.104498%	0.043343%	0.108764%	0.048109%
V13A11188	ATTACCGCGGCTGCTGGCACGTAGTTAGCCGTGGCTTCCTCCTTGGGTACCGTCA	0.016272%	0.005295%	0.045527%	0.026837%
V13A5050	ATTACCGCGGCTGCTGGCACGGAGTTAGCCGATGCTTATTCGTGCGGTACTCTCA	0.086035%	0.048021%	0.059495%	0.019942%
V13A258	ATTACCGCGGCTGCTGGCACGTAGTTAGCCGGAGCTTCCTTCTCGGGTACCGTCA	0.068369%	0.020358%	0.055459%	0.013430%
V13A8414	ATTACCGCGGCTGCTGGCACGTAGTTAGCCGTGGCTTCCTTGACAGATACCGTCA	0.060047%	0.026266%	0.063741%	0.042952%
V13A13323	ATTACCGCGGCTGCTGGCACGAAGTTTGCCGGGGCTTCTTTAGATGTTACCGTCA	0.118083%	0.070991%	0.028087%	0.011987%
V13A10709	ATTACCGCGGCTGCTGGCACGTAGTTAGCCGGAGCTTCCTCCTCAGGTACCGTCA	0.030734%	0.010855%	0.036315%	0.012108%
V13A1236	ATTACCGCGGCTGCTGGCACGTAGTTAGCCGGAGCTTATTCAAGAGGTACCGTCT	0.089530%	0.039263%	0.060709%	0.019819%
V13A8850	ATTACCGCGGCTGCTGGCACGGAGTTAGCCGATGCTTTTTCTTCGGATACTTGCA	0.019434%	0.008532%	0.074119%	0.049765%
V13A14699	ATTACCGCGGCTGCTGGCACGTATTTAGCCGGTGCTTCTTACTCAGGTACCGTCA	0.067518%	0.024450%	0.030309%	0.008587%
V13A6014	ATTACCGCGGCTGCTGGCACGTAGTTAGCCGGGGCTTCTTAGCCAGGTACCGTCA	0.040351%	0.026405%	0.052468%	0.039166%
V13A1661	ATTACCGCGGCTGCTGGCACGGAGTTAGCCGATGCTTTTTCTCCGGGTACACGCA	0.062096%	0.034851%	0.081020%	0.021781%
V13A9296	ATTACCGCGGCTGCTGGCACGTAGTTAGCCGGAGCTTCCTCCTAAGGTACCGTCA	0.043959%	0.021979%	0.019808%	0.012991%
V13A45	ATTACCGCGGCTGCTGGCACGTAGTTAGCCGGAGCTTCCTCCTTGGGTACCGTCA	0.074777%	0.053482%	0.017387%	0.004133%
V13A603	ATTACCGCGGCTGCTGGCACGTATTTAGCCGGAGCTTCTTTTTCAGGTACCGTCA	0.135898%	0.056931%	0.017670%	0.017670%
V13A1295	ATTACCGCGGCTGCTGGCACGTAGTTAGCCGGGGCTTTCTTACGAGGTACCGTCA	0.073239%	0.015167%	0.034518%	0.011023%
V13A12979	ATTACCGCGGCTGCTGGCACGTAGTTAGCCGTGGCTTACTTTACAGGTACCGTCA	0.045461%	0.017193%	0.030456%	0.013540%
V13A5114	ATTACCGCGGCTGCTGGCACGTAGTTAGCCGTGGCTTCCTTGAACGGTACCGTCA	0.074931%	0.015378%	0.042323%	0.015180%

V13A3848	ATTACCGCGGCTGCTGGCACGTAGTTAGCCGTGGCTTATTCATACAGTACCGTCA	0.030343%	0.006229%	0.035692%	0.008495%
V13A11648	ATTACCGCGGCTGCTGGCACGTAGTTAGCCGTGGCTTCCTCCTCAGGTACCGTCA	0.028438%	0.009794%	0.024223%	0.007373%
V13A12995	ATTACCGCGGCTGCTGGCACCAGACTTGCCCTCCAATGGATCCTCGTTAAGGGAT	0.023978%	0.004845%	0.012853%	0.004422%
V13A819	ATTACCGCGGCTGCTGGCACATAGTTAGCCGGGGCTTCCTCCTTAGGTACTGTCT	0.063650%	0.048275%	0.006537%	0.004800%
V13A622	ATTACCGCGGCTGCTGGCACGTAGTTAGCCGGAGCTTCCTCCTAAAGTACCGTCA	0.074626%	0.035672%	0.015386%	0.004756%
V13A12536	ATTACCGCGGCTGCTGGCACGTAGTTAGCCGTGGCTTTCTTGAATGGTACAGTCA	0.000504%	0.000504%	0.020131%	0.007809%
V13A10103	ATTACCGCGGCTGCTGGCACGTAGTTAGCCGTGGCTTATTCGACAGGTACCGTCT	0.031417%	0.009301%	0.076839%	0.060777%
V13A6899	ATTACCGCGGCTGCTGGCACGAAGTTAGCCGGGGCTTCTTTAGATGCTACCGTCA	0.041963%	0.026224%	0.015986%	0.012218%
V13A6309	ATTACCGCGGCTGCTGGCACGTAGTTAGCCGGAGCTTCCTCCCCGGGTACCGTCA	0.037062%	0.018850%	0.069357%	0.035221%
V13A7383	ATTACCGCGGCTGCTGGCACGTAGTTAGCCGATGCTTATTCTGTCGGTACTGTCA	0.053433%	0.020862%	0.019365%	0.007335%
V13A2225	ATTACCGCGGCTGCTGGCACGTAGTTAGCCGTGGCTTTCTCCTGAGGTACCATCA	0.052873%	0.030087%	0.034921%	0.019502%
V13A1207	ATTACCGCGGCTGCTGGCACGTAGTTAGCCGTGGCTTATTCCTTAGGTACCGTCA	0.028176%	0.008400%	0.052805%	0.024462%
V13A1323	ATTACCGCGGCTGCTGGCACCAGACTTGCCCTCTCATAGATCCTCGTTAACGGTT	0.049526%	0.033352%	0.029041%	0.016527%
V13A8742	ATTACCGCGGCTGCTGGCACGTAGTTAGCCGTGGCTTTCTGGCCGGGTACCATCC	0.053777%	0.020249%	0.015190%	0.004402%
V13A3151	ATTACCGCGGCTGCTGGCACGTAGTTAGCCGGGGCTTCCTCCCGGGGTACCGTCA	0.036430%	0.007472%	0.022305%	0.006058%
V13A12977	ATTACCGCGGCTGCTGGCACGTAGTTTGCCGGGGCTTTCTTACAGGGTACCGTCA	0.052417%	0.014943%	0.031909%	0.010334%
V13A5881	ATTACCGCGGCTGCTGGCACGTAGTTAGCCGTGGCTTTCTCATAAGGTACCGTCA	0.016214%	0.008069%	0.020589%	0.016465%
V13A10500	ATTACCGCGGCTGCTGGCACGTAGTTAGCCGGTGCTTTTTCTTCAGGTACAGTCA	0.078412%	0.043324%	0.006887%	0.003956%
V13A10983	ATTACCGCGGCTGCTGGCACCAGACTTGCCCTCCAATCGATTTTCTACGATGTTT	0.080918%	0.046278%	0.014534%	0.011319%
V13A898	ATTACCGCGGCTGCTGGCACGTAGTTAGCCGGAGCTTATTCTACAGGTACTGTCA	0.029731%	0.012424%	0.025222%	0.007326%
V13A15549	ATTACCGCGGCTGCTGGCACGTAGTTAGCCGTGGCTTCCTCCACAGGTACCGTCA	0.026858%	0.008490%	0.032291%	0.009624%
V13A7345	ATTACCGCGGCTGCTGGCACGTAGTTAGCCGTGGCTTCCTCATAGAGTACCGTCA	0.022928%	0.007574%	0.020358%	0.007260%
V13A891	ATTACCGCGGCTGCTGGCACGTAGTTAGCCGTGGCTTCCTCCTCGGCTACCGTCA	0.036611%	0.009178%	0.034439%	0.006284%
V13A6662	ATTACCGCGGCTGCTGGCACATAGTTAGCCGGGGCTTCCTCCTTAGGTACCGTCC	0.044603%	0.023166%	0.011930%	0.009106%
V13A9295	ATTACCGCGGCTGCTGGCACGTAGTTAGCCGGGGCTTCTTGGTCAGGTACTGTCA	0.051382%	0.027241%	0.041841%	0.018106%
V13A1755	ATTACCGCGGCTGCTGGCACGTAGTTAGCCGGAGCTTTCTTTGAGAGTACCGTCA	0.020840%	0.012361%	0.016154%	0.007961%
V13A10871	ATTACCGCGGCTGCTGGCACGTAGTTAGCCGTGGCTTCCTCTCGGGGTACCGTCA	0.016564%	0.005429%	0.041233%	0.014454%
V13A796	ATTACCGCGGCTGCTGGCACGTAGTTAGCCGGGGCTTCTTATTCAGGTACTGTCC	0.022414%	0.011375%	0.037062%	0.022371%
V13A15271	ATTACCGCGGCTGCTGGCACGTAGTTAGCCGGGGCTTTCTGGTATGGTACCATCA	0.014182%	0.003150%	0.022463%	0.007969%
V13A8834	ATTACCGCGGCTGCTGGCACGTAGTTAGCCGGGGCTTCCTCCTTATCTACCGTCC	0.013736%	0.003296%	0.025045%	0.006099%

V13A4197	ATTACCGCGGCTGCTGGCACGTAGTTAGCCGGGGCTTCCTCCTATGGTACCGTCA	0.019243%	0.006796%	0.012163%	0.003426%
V13A5096	ATTACCGCGGCTGCTGGCACGTAGTTAGCCGGAGCTTTCTTGTAAGGTACCGTCA	0.036034%	0.020671%	0.010242%	0.006379%
V13A43	ATTACCGCGGCTGCTGGCACGTAGTTAGCCGGAGCTTCCTCCTTGGGTACCGTCC	0.023751%	0.005714%	0.034562%	0.011650%
V13A5077	ATTACCGCGGCTGCTGGCACGTAGTTAGCCGGGGCTTCTTGGTCGGGTACCGTCT	0.035028%	0.010758%	0.030455%	0.014709%
V13A5836	ATTACCGCGGCTGCTGGCACGTAGTTAGCCGTGGCTTATTCATCAAGTACCGTCA	0.025025%	0.013283%	0.020907%	0.005675%
V13A10710	ATTACCGCGGCTGCTGGCACGTAGTTAGCCGGAGCTTATTCATCAGGTACCGTCA	0.017525%	0.005941%	0.024884%	0.005484%
V13A10997	ATTACCGCGGCTGCTGGCACGTAGTTAGCCGGGGCTTCTTAGTCAAGTACCGTCA	0.011053%	0.005464%	0.012499%	0.007432%
V13A4643	ATTACCGCGGCTGCTGGCACGTAGTTAGCCGGGGCTTATTCAAATGGTACCGTCA	0.009602%	0.004410%	0.028518%	0.010199%
V13A440	ATTACCGCGGCTGCTGGCACGGAATTAGCCGGTCCTTATTCATAATGTACATGCA	0.007655%	0.003586%	0.037001%	0.013589%
V13A6197	ATTACCGCGGCTGCTGGCACGTAGTTAGCCGTGGCTTGCTCCTTAGCTACCGTCA	0.015500%	0.005806%	0.020753%	0.011307%
V13A6942	ATTACCGCGGCTGCTGGCACGTAGTTAGCCGTGACTTTCTCACAAGGTACCGTCA	0.034621%	0.016264%	0.019691%	0.005559%
V13A5387	ATTACCGCGGCTGCTGGCACGTAGTTAGCCGTGACTTTCTCACAGGGTACCGTCA	0.034370%	0.013810%	0.011291%	0.005045%
V13A6299	ATTACCGCGGCTGCTGGCACGTAGTTAGCCGGGGCTTCCTCCTTAGGTACTGTCC	0.025375%	0.008070%	0.007418%	0.003443%
V13A2809	ATTACCGCGGCTGCTGGCACGTAGTTAGCCGGGGCTTCTTACTCAGGTACCGTCC	0.009345%	0.004964%	0.039442%	0.021069%
V13A2594	ATTACCGCGGCTGCTGGCACGTAGTTAGCCGGGGCTTTCTGGTAAAGTACCGTCA	0.039965%	0.016541%	0.015578%	0.006088%
V13A13703	ATTACCGCGGCTGCTGGCACGTAGTTAGCCGGGGCTTCTTACTCAGGTACTGTCT	0.026908%	0.011801%	0.032807%	0.019146%
V13A6109	ATTACCGCGGCTGCTGGCACGTAGTTAGCCGTGGCTTTCTATTCCGGTACCGTCA	0.008010%	0.004364%	0.030545%	0.017169%
V13A10328	ATTACCGCGGCTGCTGGCACGTAGTTAGCCGGAGCTTCCTCTTTGGGTACCGTCC	0.024979%	0.009250%	0.027026%	0.007568%
V13A9008	ATTACCGCGGCTGCTGGCACTGAATTAGCCGGTCCTTATTCATAAGGTACATGCA	0.007492%	0.003282%	0.024375%	0.006770%
V13A8732	ATTACCGCGGCTGCTGGCACGTAGTTAGCCGTGGCTTATTCGAGGGGTACCGTCT	0.018118%	0.006179%	0.035035%	0.011855%
V13A11770	ATTACCGCGGCTGCTGGCACGTAGTTAGCCGGAGCTTTCTTGCAGGGTACCGTCA	0.023739%	0.008282%	0.021476%	0.008118%
V13A12847	ATTACCGCGGCTGCTGGCACGTAGTTAGCCGTGGCTTCTTCTGCAGGTACCGTCC	0.019730%	0.008034%	0.005424%	0.002490%
V13A1325	ATTACCGCGGCTGCTGGCACGTAGTTAGCCGTGGCTTTCTTATAGAGTACCGTCA	0.025109%	0.015889%	0.006048%	0.003057%
V13A8060	ATTACCGCGGCTGCTGGCACGTAGTTAGCCGGAGCTTATTCGACAGGTACCGTCT	0.025726%	0.009599%	0.018886%	0.005481%
V13A1489	ATTACCGCGGCTGCTGGCACGTAGTTAGCCGTGGCTTATTCCTATGGTACCGTCA	0.014739%	0.004344%	0.029426%	0.012108%
V13A2667	ATTACCGCGGCTGCTGGCACGGAGTTAGCCGATCCTTATTCGTACTATACTTTCA	0.010758%	0.004299%	0.015782%	0.007241%
V13A9569	ATTACCGCGGCTGCTGGCACGTAGTTAGCCGGGGCTTCTTATTTAGGTACCGTCA	0.011880%	0.003198%	0.035201%	0.006789%
V13A6720	ATTACCGCGGCTGCTGGCACGTAGTTAGCCGGAGCTTATTCTACAGGTACCGTCA	0.031759%	0.013225%	0.004635%	0.003001%
V13A14908	ATTACCGCGGCTGCTGGCACGTAGTTAGCCGGGGCTTCCTCCTAAGATACCGTCT	0.012608%	0.006515%	0.013748%	0.004029%
V13A4443	ATTACCGCGGCTGCTGGCACGTAGTTAGCCGTGACTTTCTGGCGGGGCACCATCA	0.007076%	0.003472%	0.014894%	0.004772%

V13A14335	ATTACCGCGGCTGCTGGCACGTAGTTAGCCGGGACTTCTTAGTCAGGTACCGTCT	0.018661%	0.006413%	0.010476%	0.003577%
V13A5932	ATTACCGCGGCTGCTGGCACGTAGTTAGCCGTGGCTTATTCGTTGAGTACCGTCA	0.013105%	0.005328%	0.010479%	0.003787%
V13A13046	ATTACCGCGGCTGCTGGCACGTAGTTAGCCGTGGCTTCCTCTTTAGCTACCGTCA	0.012333%	0.006379%	0.002226%	0.001167%
V13A4925	ATTACCGCGGCTGCTGGCACGGAGTTAGCCGGTGCTTCCTTTGGAGGTACCGTCA	0.016434%	0.006657%	0.015510%	0.009006%
V13A12537	ATTACCGCGGCTGCTGGCACGTAGTTAGCCGTGGCTTTCTCCTATGGTACCGTCA	0.015953%	0.007490%	0.013838%	0.004103%
V13A14350	ATTACCGCGGCTGCTGGCACGGAGTTAGCCGATGCTTATTCATAAGGTACAGGCA	0.008372%	0.003558%	0.013195%	0.005612%
V13A8893	ATTACCGCGGCTGCTGGCACGTAGTTAGCCGGAGCTTTCTTCACAGGTACAGTCT	0.021960%	0.007652%	0.004778%	0.002655%
V13A8818	ATTACCGCGGCTGCTGGCACGTAGTTAGCCGGGGCTTCTTCTGCAGGTACCGTCA	0.024806%	0.015418%	0.005160%	0.003560%
V13A7459	ATTACCGCGGCTGCTGGCACGTAGTTAGCCGTGGCTTATTCTTACGGTACCGTCA	0.021019%	0.004235%	0.010870%	0.003210%
V13A8406	ATTACCGCGGCTGCTGGCACGGAATTAGCCAGTCCTTATTCATGCGGTACCTGCA	0.036337%	0.033393%	0.002346%	0.001583%
V13A646	ATTACCGCGGCTGCTGGCACGTAGTTAGCCGGGGCTTCTTATTCGGGTACTGTCA	0.021769%	0.011788%	0.010363%	0.004330%
V13A7019	ATTACCGCGGCTGCTGGCACGTAGTTAGCCGGGGCTTCCTTCTTGGCTACCATCA	0.000000%	0.000000%	0.007675%	0.003669%
V13A10250	ATTACCGCGGCTGCTGGCACGTAGTTAGCCGTGGCTTCCTCATAGGGTACCGTCA	0.017992%	0.005289%	0.011708%	0.003291%
V13A6419	ATTACCGCGGCTGCTGGCACGTAGTTAGCCGTGGCTTCCTTCCCCGCTACCGTCA	0.004748%	0.001787%	0.006222%	0.003641%
V13A12462	ATTACCGCGGCTGCTGGCACGTAGTTAGCCGGGGCTTATTCAAAAGGTACCGTCA	0.015194%	0.003495%	0.006856%	0.002210%
V13A5524	ATTACCGCGGCTGCTGGCACGTAGTTAGCCGGAGCTTATTCTACAGGTACTGTCC	0.004238%	0.002163%	0.012738%	0.009043%
V13A6490	ATTACCGCGGCTGCTGGCACGTAGTTAGCCGTGGCTTCCTCCTTGGCTACCGTCA	0.018236%	0.007099%	0.004117%	0.002450%
V13A12317	ATTACCGCGGCTGCTGGCACGTATTTAGCCGGAGCTTCTTCTTCAGGTACCGTCA	0.023549%	0.012570%	0.004609%	0.004609%
V13A1324	ATTACCGCGGCTGCTGGCACGTATTTAGCCGGGGCTTCTTACTCAGGTACCGTCA	0.023069%	0.021554%	0.006115%	0.002008%
V13A14079	ATTACCGCGGCTGCTGGCACGTAGTTAGCCGTGACTTGCTTGAAGAGTACCGTCA	0.013363%	0.006927%	0.003983%	0.002425%
V13A1442	ATTACCGCGGCTGCTGGCACATAGTTAGCCGGAGCTTCCTCCTTAGGTACTGTCA	0.023523%	0.005609%	0.001454%	0.000962%
V13A9454	ATTACCGCGGCTGCTGGCACGTAGTTAGCCGTGGCTTATTCCTAAGGTACCGTCG	0.009082%	0.003283%	0.009046%	0.005058%
V13A921	ATTACCGCGGCTGCTGGCACGGAGTTAGCCGATGCTTATTCATATGGTACATACA	0.005437%	0.002401%	0.007091%	0.005284%
V13A10879	ATTACCGCGGCTGCTGGCACGTAGTTAGCCGTGGCTTTCTTTACAGGTACCGTCA	0.004443%	0.002778%	0.003568%	0.001188%
V13A15163	ATTACCGCGGCTGCTGGCACGTAGTTTGCCGGGGCTTCCTCATTAGGTACCGTCT	0.014180%	0.007245%	0.001151%	0.000774%
V13A4944	ATTACCGCGGCTGCTGGCACAGAGTTAGCCGTCTCTTCCTCTTGTGGTACTATCT	0.005727%	0.002840%	0.004793%	0.003255%
V13A12243	ATTACCGCGGCTGCTGGCACGTATTTAGCCGGGGCTTCTTAGTCAGGTACCGTCT	0.010962%	0.003740%	0.001745%	0.001158%
V13A13994	ATTACCGCGGCTGCTGGCACGTAGTTAGCCGGGGCTTCTTGGTCAGGTACCGTCG	0.007742%	0.002778%	0.002285%	0.001145%

ESM Table 3. Effects of vertical sleeve gastrectomy (VSG) and sham operation in the diabetic Goto-Kakizaki rat on plasma bile acid concentration. Mass spectrometry method was used to determine the plasma concentration of bile acids. Data are means ± SEM.

	VSG	Sham	P
BA01 Ursocholanic acid (nmol/l)	196.70 ± 14.20	141.76 ± 13.56	0.018
BA04 3-Ketocholanic acid (nmol/l)	2.00 ± 0.92	1.28 ± 0.59	0.521
BA06 Isolithocholic acid (nmol/l)	7.65 ± 1.59	7.44 ± 0.97	0.913
BA15 8(14),(5ß)-Cholenic acid-3a, 12a-diol (nmol/l)	5.60 ± 1.93	2.00 ± 0.28	0.110
BA16 5ß-Cholenic acid-7a-ol-3-one (nmol/l)	52.85 ± 33.36	12.96 ± 4.30	0.271
BA17 5a-Cholanic acid-3a-ol-6-one (nmol/l)	71.80 ± 31.37	31.60 ± 11.68	0.260
BA19 3a-hydroxy-12 ketolithocholic acid (5ß-cholanic acid-3a-ol-12-one) (nmol/l)	50.55 ± 23.87	19.84 ± 6.85	0.251
BA20 Murocholic acid (nmol/l)	81.90 ± 54.15	13.36 ± 6.05	0.251
BA22 5ß-Cholanic acid-3ß, 12a-diol (nmol/l)	23.75 ± 14.95	5.84 ± 4.90	0.293
BA23 Deoxycholic acid (nmol/l)	387.00 ± 202.43	106.96 ± 51.67	0.216
BA24 Chenodeoxycholic acid (nmol/l)	1798.1 ± 527.47	1113.76 ± 317.88	0.287
BA25 Hyodeoxycholic acid (nmol/l)	173.25 ± 71.84	78.96 ± 19.03	0.241
BA29 5ß-Cholanic acid-3a, 6a-diol-7-one (nmol/l)	94.90 ± 31.35	75.84 ± 14.73	0.591
BA30 3-Dehydrocholic acid (nmol/l)	94.00 ± 31.20	74.96 ± 14.71	0.589
BA31 12--Dehydrocholic acid (nmol/l)	482.35 ± 183.57	366.64 ± 128.07	0.621
BA32 Ursodeoxycholic acid (nmol/l)	36.45 ± 23.53	11.12 ± 8.88	0.340
BA33 omega-Muricholic acid (nmol/l)	734.30 ± 264.92	454.4 ± 135.18	0.371
BA34 ß-Muricholic acid (nmol/l)	1005.15 ± 382.91	570.56 ± 130.72	0.312
BA35 Cholic acid (nmol/l)	6558.80 ± 1584.18	6336 ± 1209.34	0.905
BA36 Hyocholic acid (nmol/l)	36.40 ± 9.71	18.08 ± 5.73	0.129
BA37 alpha-Muricholic acid (nmol/l)	429.15 ± 153.49	205.28 ± 64.19	0.210
BA39 Glycolithocholic acid (5ß-Cholanic acid-3a-ol N-N-(carboxymethyl)-amide) (nmol/l)	1.10 ± 0.20	1.04 ± 0.09	0.791
BA40 Glycohyodeoxycholic acid (5ß-Cholanic acid-3a, 6a-diol N-N-(carboxymethyl)- (nmol/l)	80.95 ± 41.78	29.04 ± 13.46	0.271
BA41 Glycochenodeoxycholic acid (nmol/l)	32.70 ± 13.82	18.16 ± 6.64	0.369
BA42 Glycodeoxycholic acid (nmol/l)	44.80 ± 26.55	30.48 ± 12.88	0.637
BA43 Glycoursodeoxycholic acid (5ß-Cholanic acid-3a,7ß-diol-N-(carboxymethyl) (nmol/l)	81.30 ± 41.91	29.76 ± 13.78	0.266
BA47 Tauro-ursocholanic acid (5ß-Cholanic acid N-(2-sulphoethyl)-amide) (nmol/l)	6.45 ± 0.24	6.48 ± 0.43	0.952
BA49 Glycohyocholic acid (nmol/l)	177.50 ± 58.55	93.28 ± 46.99	0.291
BA50 Tauro-ursodeoxycholic acid (5ß-Cholanic acid-3a,7ß-diol N-(2-sulphoethyl)- (nmol/l)	227.80 ± 78.80	35.76 ± 20.74	0.046
BA58 Tauro omega-Muricholic acid sodium salt (nmol/l)	118.30 ± 26.23	157.12 ± 111.61	0.747
BA59 Taurocholic acid (nmol/l)	403.70 ± 106.16	167.30 ± 42.17	0.069
BA48 Taurolithocholic acid (5B-Cholanic acid-3a-ol-N-(2-sulphoethyl)-amide) (nmol/l)	1.75 ± 0.35	0.88 ± 0.20	0.051

BA51 Taurohyodeoxycholic acid(5B-Cholanic acid-3a,6a-diol N-(2-sulphoethyl)-amide (nmol/l)	187.45 ± 66.75	30.24 ± 16.83	0.049
BA52 Taurochenodeoxycholic acid (nmol/l)	116.70 ± 36.27	83.92 ± 49.86	0.612
BA53 Taurodeoxycholic acid (nmol/l)	78.65 ± 34.27	10.72 ± 6.20	0.091
Total bile acids (nmol/l)	13881.80 ± 3837.74	10309.36 ± 1938.03	0.575
Primary bile acids (nmol/l)	10164.75 ± 2585.67	8507.28 ± 1503.95	0.771
Primary bile acids (cholic acid, CA) (nmol/l)	7271.30 ± 1707.79	6657.04 ± 1264.47	0.834
Primary bile acids (chenodeoxycholic acid, CDCA) (nmol/l)	1947.50 ± 562.52	1215.84 ± 289.27	0.355
Primary bile acids (muricholic acid, MCAb) (nmol/l)	1123.45 ± 403.09	727.68 ± 116.74	0.468
Secondary bile acids (nmol/l)	1310.80 ± 527.12	472.00 ± 164.15	0.249
Secondary bile acids (deoxycholic acid, DCA) (nmol/l)	871.15 ± 382.50	257.36 ± 102.16	0.244
Secondary bile acids (lithocholic acid, LCA) (nmol/l)	10.50 ± 2.08	9.36 ± 1.14	0.694
Secondary bile acids (muricholic acid, MCAa) (nmol/l)	429.15 ± 153.49	205.28 ± 64.19	0.294
Ursoconjugated (nmol/l)	345.55 ± 136.45	76.64 ± 41.83	0.160
Glycoconjugated (nmol/l)	159.90 ± 82.18	79.44 ± 33.00	0.475
Tauroconjugated (nmol/l)	1134.35 ± 331.55	452.48 ± 138.86	0.154
MCA/CA	0.15 ± 0.02	0.12 ± 0.02	0.382
Primary/Secondary	9.47 ± 1.51	26.31 ± 8.24	0.033

ESM Table 4. Effects of enhanced P. copri in the gut microbiota of diabetic Goto Kakizaki rats on the plasma levels of bile acids. Mass spectrometry method was used to determine the plasma concentration of bile acids in Goto-Kakizaki rats inoculated with gut microbiota (GM) from sleeve operated and sham GK, treated with a mixture of kanamycin and vancomycin antibiotics or inoculated with *Prevotella Copri*. ND, Not Detected. Data are means ± SEM.

	GM Transfer	Controls	P	Antibiotics	Controls	P	P. Copri	Controls	P
Lithocholic acid (nmol/l)	20.00 ± 5.09	6.18 ± 0.59	0.053	4.36 ± 0.17	16.37 ± 1.05	0.006	12.18 ± 1.81	10.90 ± 4.97	0.827
BA01 Ursocholanic acid (nmol/l)	27.70 ± 3.73	14.28 ± 2.88	0.025	7.88 ± 0.89	10.23 ± 0.66	0.077	10.70 ± 1.04	7.93 ± 1.97	0.298
5-Cholenic acid-3ß-ol (nmol/l)	0.42 ± 0.12	0.23 ± 0.02	0.193	0.22 ± 0.04	0.40 ± 0.06	0.064	0.62 ± 0.15	0.37 ± 0.15	0.276
Lithocholenic acid (nmol/l)	2.16 ± 1.78	1.78 ± 0.05	0.119	1.48 ± 0.11	1.67 ± 0.09	0.227	1.78 ± 0.10	1.60 ± 0.15	0.376
BA06 Isolithocholic acid (nmol/l)	1.92 ± 0.27	0.55 ± 0.10	0.005	1.46 ± 0.11	1.43 ± 0.23	0.924	2.03 ± 0.50	2.00 ± 0.42	0.961
3a,12a, 23-Nordeoxycholic acid (nmol/l)	2.06 ± 0.09	2.23 ± 0.15	0.390	1.60 ± 0.03	2.43 ± 0.30	0.105	2.10 ± 0.04	2.13 ± 0.17	0.863
BA17 5a-Cholanic acid-3a-ol-6-one (nmol/l)	22.92 ± 8.63	14.83 ± 6.47	0.478	ND	29.43 ± 4.92	0.027	79.27 ± 33.23	29.87 ± 19.09	0.239
BA19 3a-hydroxy-12 ketolithocholic acid (5ß-cholanic acid-3a-ol-12-one) (nmol/l)	7.40 ± 3.44	2.85 ± 0.81	0.261	0.10 ± 0.00	2.27 ± 0.22	0.010	6.97 ± 1.92	3.83 ± 0.58	0.170
BA20 Murocholic acid (nmol/l)	5.50 ± 3.14	1.90 ± 0.68	0.317	0.02 ± 0.02	3.47 ± 0.78	0.048	28.83 ± 13.97	5.33 ± 1.84	0.154
BA22 5ß-Cholanic acid-3ß, 12a-diol (nmol/l)	7.98 ± 4.02	1.08 ± 0.78	0.162	ND	1.60 ± 0.06	0.001	3.85 ± 2.05	5.73 ± 3.39	0.662
BA23 Deoxycholic acid (nmol/l)	92.3 ± 42.67	23.65 ± 5.99	0.184	5.02 ± 0.12	22.07 ± 3.23	0.034	58.20 ± 24.91	46.67 ± 8.28	0.676
BA24 Chenodeoxycholic acid (nmol/l)	198.14 ± 143.49	41.93 ± 22.48	0.340	9.34 ± 2.31	139.43 ± 35.65	0.067	528.22 ± 116.52	154.77 ± 99.72	0.048
BA25 Hyodeoxycholic acid (nmol/l)	222.76 ± 93.13	99.10 ± 42.80	0.277	ND	136.23 ± 35.77	0.063	658.70 ± 240.34	191.63 ± 100.94	0.120
BA29 5ß-Cholanic acid-3a, 6a-diol-7-one (nmol/l)	41.3 ± 29.64	11.18 ± 7.53	0.375	1.02 ± 0.87	37.37 ± 7.20	0.035	181.83 ± 65.19	56.33 ± 33.93	0.145
BA30 3-Dehydrocholic acid (nmol/l)	9.38 ± 8.70	0.68 ± 0.69	0.374	ND	0.10 ± 0.10	0.423	14.37 ± 6.36	2.87 ± 2.87	0.146
BA31 12--Dehydrocholic acid (nmol/l)	83.1 ± 76.49	8.10 ± 4.81	0.383	12.84 ± 11.82	154.97 ± 73.76	0.191	567.87 ± 417.97	46.03 ± 21.48	0.267
BA32 Ursodeoxycholic acid (nmol/l)	25.22 ± 18.27	6.15 ± 3.23	0.359	0.82 ± 0.22	38.30 ± 16.41	0.150	64.37 ± 21.62	17.03 ± 6.28	0.082
BA33 omega-Muricholic acid (nmol/l)	80.9 ± 54.53	28.18 ± 20.38	0.406	1.28 ± 0.88	41.97 ± 9.00	0.044	235.20 ± 67.04	29.35 ± 8.45	0.027
BA35 Cholic acid (nmol/l)	562 ± 349.06	275.48 ± 210.42	0.507	9.42 ± 5.13	276.03 ± 72.61	0.066	1130.22 ± 213.96	217.60 ± 20.20	0.008
BA39 Glycolithocholic acid (5ß-Cholanic acid-3a-ol N-N-(carboxymethyl)-amide) (nmol/l)	0.56 ± 0.06	0.53 ± 0.02	0.612	0.52 ± 0.02	0.50 ± 0.01	0.374	0.68 ± 0.07	0.50 ± 0.06	0.095
BA40 Glycohyodeoxycholic acid (5ß-Cholanic acid-3a, 6a-diol N-N-(carboxymethyl) (nmol/l)	4.18 ± 2.18	2.00 ± 1.55	0.444	ND	14.37 ± 2.78	0.036	23.68 ± 12.23	15.90 ± 14.20	0.695

BA43 Glycoursodeoxycholic acid (5ß-Cholanic acid-3a,7ß-diol-N-(carboxymethyl) (nmol/l)	0.40 ± 0.13	0.23 ± 0.06	0.274	0.26 ± 0.02	0.50 ± 0.12	0.168	0.63 ± 0.17	0.37 ± 0.12	0.236
BA47 Tauro-ursocholanic acid (5ß-Cholanic acid N-(2-sulphoethyl)-amide) (nmol/l)	0.94 ± 0.13	0.38 ± 0.11	0.013	0.36 ± 0.04	0.47 ± 0.03	0.088	0.40 ± 0.04	0.30 ± 0.06	0.223
BA50 Tauro-ursodeoxycholic acid (5ß-Cholanic acid-3a,7ß-diol N-(2-sulphoethyl) (nmol/l)	1.30 ± 0.40	0.93 ± 0.25	0.457	1.80 ± 0.52	0.87 ± 0.37	0.194	0.80 ± 0.15	0.50 ± 0.21	0.303
BA58 Tauro omega-Muricholic acid sodium salt (nmol/l)	34.06 ± 3.29	29.25 ± 5.58	0.491	43.82 ± 8.50	30.67 ± 13.02	0.448	5.62 ± 0.59	6.47 ± 3.43	0.829
BA59 Taurocholic acid (nmol/l)	54.28 ± 11.01	57.75 ± 11.92	0.837	148.2 ± 22.19	76.87 ± 34.73	0.165	11.93 ± 1.84	15.67 ± 6.00	0.603
Allolithocholic acid (nmol/l)	0.24 ± 0.12	0.35 ± 0.18	0.622	0.18 ± 0.04	2.60 ± 0.23	0.008	0.77 ± 0.17	0.17 ± 0.07	0.016
Total bile acids (nmol/l)	1509.14 ± 851.83	631.7 ± 323.38	0.379	252.00 ± 39.78	1042.60 ± 214.42	0.062	3631.82 ± 1087.17	789.53 ± 173.30	0.047
Primary bile acids (nmol/l)	889.78 ± 532.15	415.58 ± 236.84	0.450	211.80 ± 32.53	560.37 ± 106.70	0.071	1857.82 ± 372.38	378.30 ± 71.14	0.010
Primary bile acids (cholic acid, CA) (nmol/l)	657.58 ± 388.14	344.40 ± 216.37	0.507	158.64 ± 24.25	390.27 ± 74.36	0.077	1323.98 ± 268.46	217.07 ± 40.21	0.009
Primary bile acids (chenodeoxycholic acid, CDCA) (nmol/l)	198.14 ± 143.49	41.93 ± 22.48	0.340	9.34 ± 2.31	139.43 ± 35.65	0.067	528.22 ± 116.52	154.77 ± 99.72	0.048
Primary bile acids (muricholic acid, MCAb) (nmol/l)	34.06 ± 3.29	29.25 ± 5.58	0.491	43.82 ± 8.50	30.67 ± 13.02	0.448	5.62 ± 0.59	6.47 ± 3.43	0.824
Secondary bile acids (nmol/l)	337.54 ± 139.47	130.00 ± 49.01	0.220	11.36 ± 0.22	176.60 ± 38.46	0.050	731.80 ± 259.99	251.70 ± 98.06	0.133
Secondary bile acids (Deoxycholic acid, DCA) (nmol/l)	315.06 ± 134.76	122.75 ± 48.76	0.237	5.02 ± 0.12	158.30 ± 37.69	0.055	716.90 ± 258.51	238.30 ± 92.70	0.131
Secondary bile acids (lithocholic acid, LCA) (nmol/l)	2.48 ± 0.28	1.08 ± 0.11	0.005	1.98 ± 0.10	1.93 ± 0.23	0.867	2.72 ± 0.56	2.50 ± 0.47	0.777
Ursoconjugated (nmol/l)	26.92 ± 18.80	7.30 ± 3.11	0.359	2.88 ± 0.61	39.67 ± 16.88	0.161	65.80 ± 21.90	17.90 ± 6.27	0.082
Glycoconjugated (nmol/l)	0.96 ± 0.19	0.75 ± 0.06	0.341	0.78 ± 0.04	1.00 ± 0.12	0.189	1.32 ± 0.24	0.87 ± 0.18	0.171
Tauroconjugated (nmol/l)	89.64 ± 13.30	87.93 ± 17.46	0.940	193.82 ± 30.96	108.40 ± 48.11	0.216	18.35 ± 2.20	22.63 ± 7.68	0.639
MCA/CA	0.19 ± 0.09	0.19 ± 0.07	0.940	0.27 ± 0.02	0.08 ± 0.03	0.006	0.01 ± 0.00	0.03 ± 0.01	0.203
Primary/Secondary	2.01 ± 0.49	2.67 ± 0.49	0.379	18.88 ± 3.26	3.42 ± 0.89	0.007	6.21 ± 2.90	1.92 ± 073	0.205

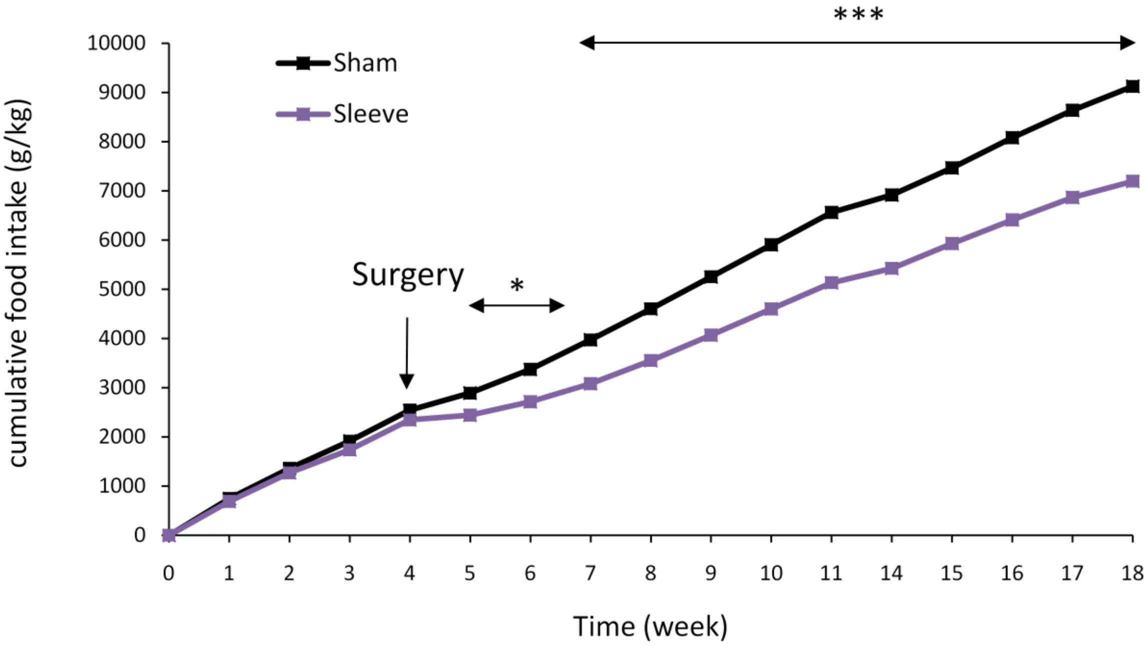

ESM Figure 1. Food intake in Goto-Kakizaki (GK) rats following vertical sleeve gastrectomy (VSG, Sleeve) or sham operation. *P<0.05, ***P<0.001 significant differences between VSG rats and sham controls.

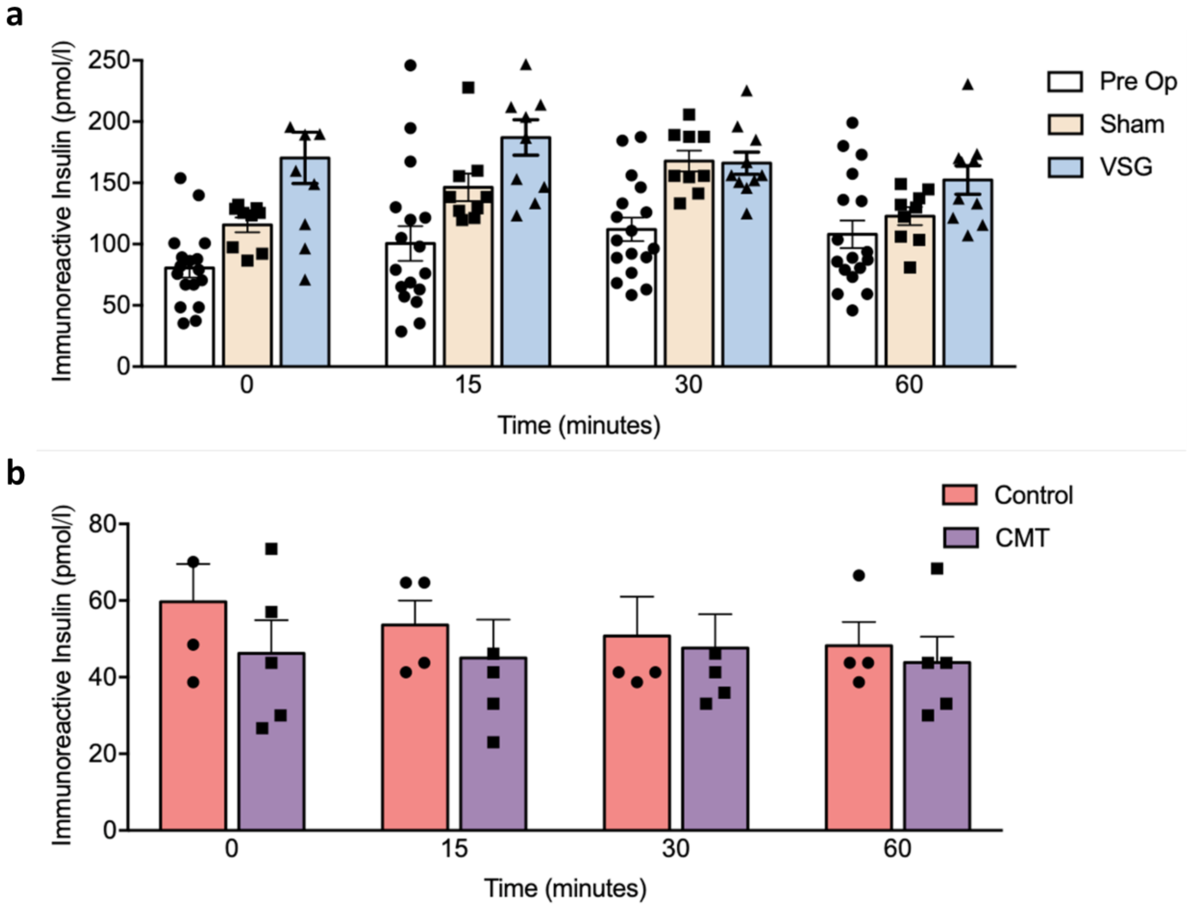

ESM Figure 2. Effects of vertical sleeve gastrectomy and gut microbiota transfer from gastrectomised Goto-Kakizaki (GK) rats on insulin secretion. Glucose-induced insulin secretion was determined in GK rats following vertical sleeve gastrectomy (VSG) or sham operation (a) and following inoculation of GK rats with gut microbiota from gastrectomised GK rats or sham controls (b). Insulin secretion tests (see methods) were performed following an overnight (16h) fast before VSG (Pre Op) and 91 days after VSG (n=10) or sham operation (n=9) (a), and 12 days after transfer of caecal microbiota transfer (CMT) from VSG treated GK rats (n=5) or sham operated GK rats (n=4) (b). Data are mean ±SEM.

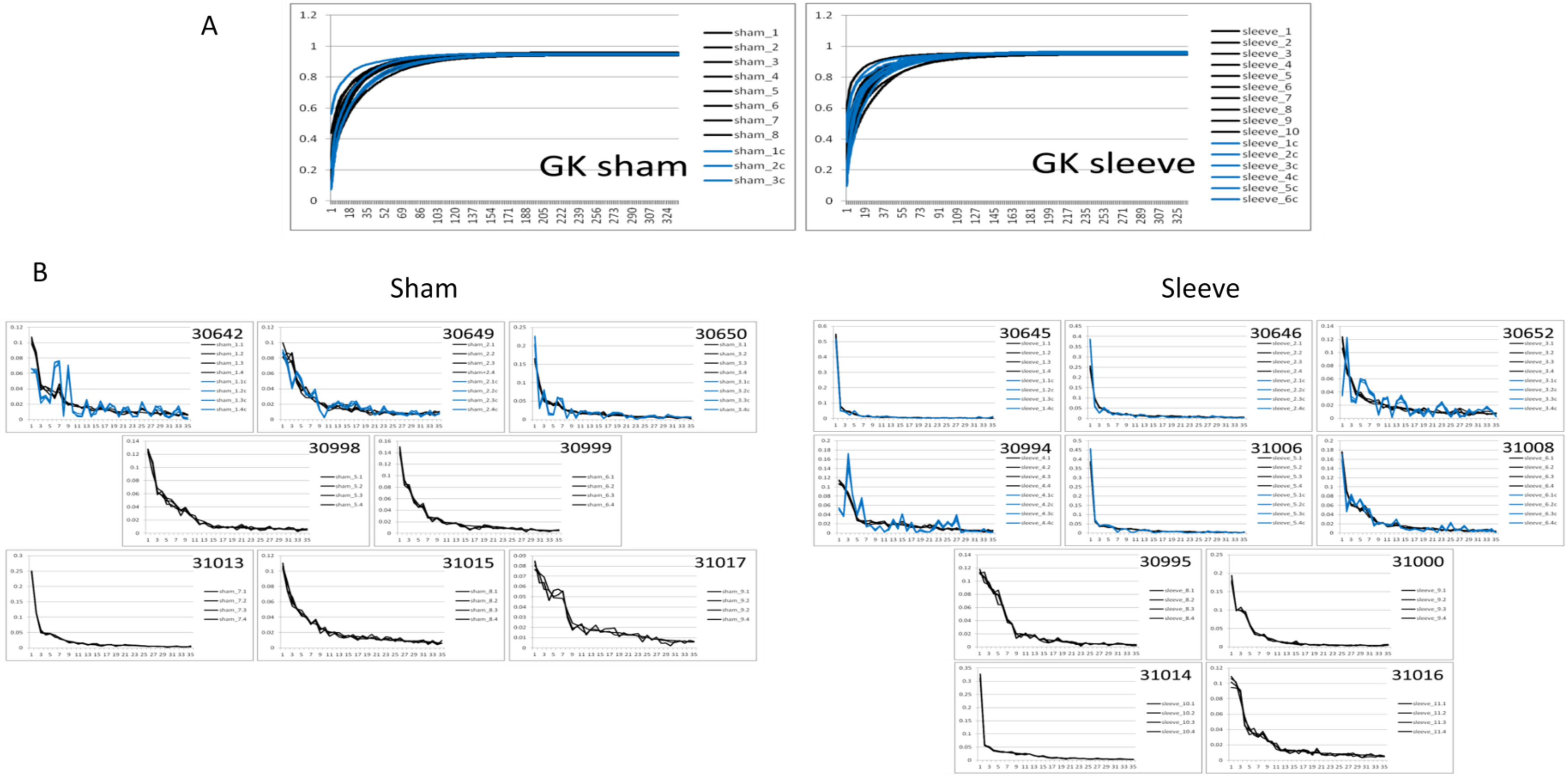

ESM Figure 3. Saturation curves for biological replicates (A) and technical reproducibility (B) of sequencing data in gastrectomised (vertical sleeve gastrectomy) or sham operated Goto-Kakizaki rats. Technical reproducibility was assessed across the 4 different sequencing runs for each caecum and colon sample. Data were sorted by the caecum first to 35st most common 16S 55mers. Data from caecum and colon are shown in black and blue lines, respectively.

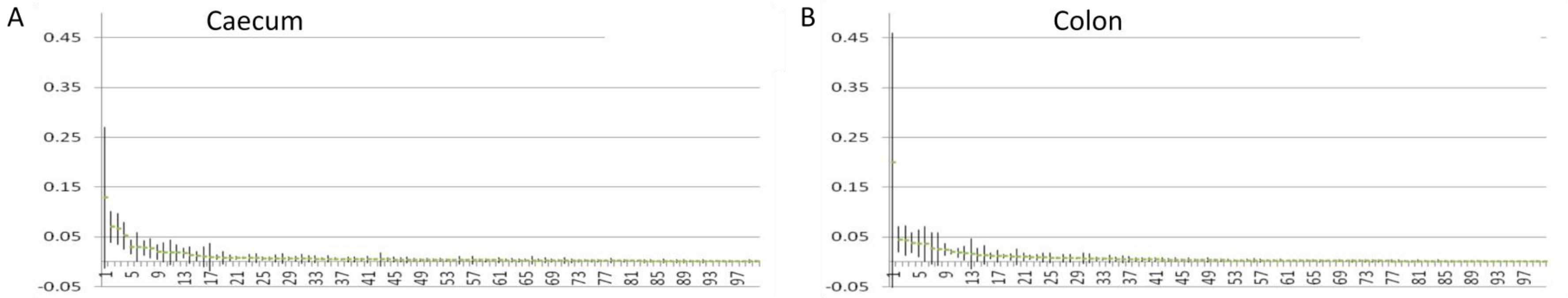

C

Caecum							Colon			
30649	30650	30998	30999	31013	31015	31017	30642	30649	30650	
0.28	0.07	0.28	0.12	0.03	0.15	0.35	0.61	0.27	<0.01	30642
	0.15	0.31	0.20	0.15	0.53	0.62	0.22	0.90	0.04	30649
		0.37	0.80	0.72	0.53	0.23	0.08	0.18	0.81	30650
			0.55	0.37	0.42	0.38	0.14	0.38	0.19	30998
				0.73	0.58	0.27	0.06	0.24	0.59	30999
					0.61	0.15	0.04	0.19	0.73	31013
						0.15	0.14	0.65	0.31	31015
							0.46	0.70	0.08	31017
								0.25	0.04	30642
									0.06	30649

ESM Figure 4. Reproducibility of sequencing data among biological replicates in sham operated Goto-Kakizaki rats. Frequencies of the 100 most common 16S-rDNA sequences are plotted for caecum (A) and colon (B) datasets. Mean and standard deviation illustrate sequence data variability. The R-squared value of each biological replicate comparison was calculated to evaluate consistency of sequencing outputs between colon samples, between caecum samples and between colon and caecum samples (C). Background color in C indicates comparisons between different animals (Grey, caecum vs caecum; Blue, colon vs colon; Light green, caecum vs colon) or for the same animals (Darker green, caecum vs colon). Red text underlines R-squared value >0.75.

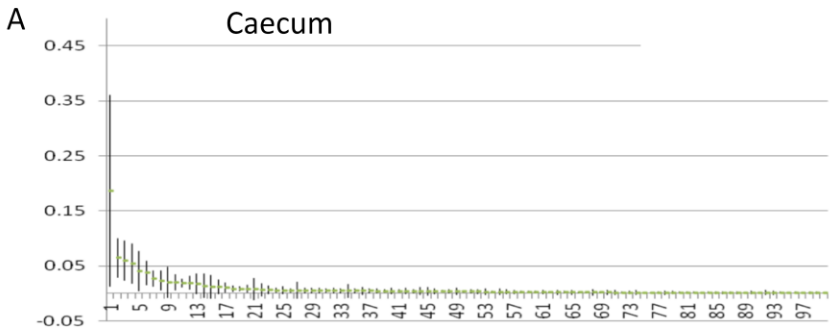

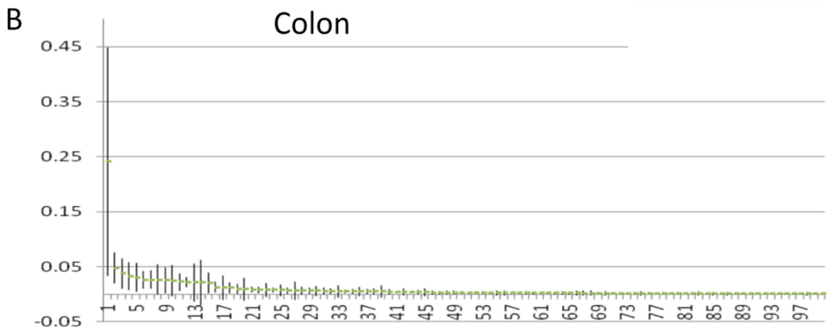

C

Caecum									Colon						
30646	30652	30994	31006	31008	30995	31000	31014	31016	30645	30646	30652	30994	31006	31008	
0.77	<0.01	0.17	0.91	0.57	0.21	0.49	0.89	<0.01	0.99	0.92	0.01	0.57	0.93	0.54	30645
	0.08	0.55	0.83	0.73	0.46	0.73	0.91	0.13	0.78	0.93	0.07	0.73	0.79	0.76	30646
		0.45	0.01	0.14	0.33	0.18	0.04	0.56	<0.01	0.01	0.48	0.08	<0.01	0.17	30652
			0.26	0.48	0.55	0.51	0.39	0.49	0.18	0.33	0.24	0.57	0.20	0.57	30994
				0.71	0.33	0.62	0.96	0.02	0.92	0.94	0.02	0.67	0.99	0.67	31006
					0.79	0.90	0.78	0.28	0.58	0.68	0.12	0.65	0.66	0.93	31008
						0.84	0.43	0.60	0.21	0.32	0.18	0.47	0.27	0.81	30995
							0.72	0.43	0.49	0.62	0.12	0.54	0.55	0.92	31000
								0.07	0.90	0.95	0.03	0.73	0.93	0.76	31014
									<0.01	0.03	0.20	0.09	0.01	0.35	31016
										0.92	0.01	0.57	0.94	0.54	30645
											0.04	0.70	0.93	0.67	30646
												0.17	0.01	0.13	30652
													0.64	0.70	30994
														0.61	31006

ESM Figure 5. Reproducibility of sequencing data among biological replicates in gastrectomised (sleeve) Goto-Kakizaki rats. Frequencies of the 100 most common 16S-rDNA sequences are plotted for caecum (A) and colon (B) datasets. Mean and standard deviation illustrate sequence data variability. The R-squared value of each biological replicate comparison was calculated to evaluate consistency of sequencing outputs between colon samples, between caecum samples and between colon and caecum samples (C). Background color in C indicates comparisons between different animals (Grey, caecum vs caecum; Blue, colon vs colon; Light green, caecum vs colon) or for the same animals (Darker green, caecum vs colon). Red text underlines R-squared value >0.75.

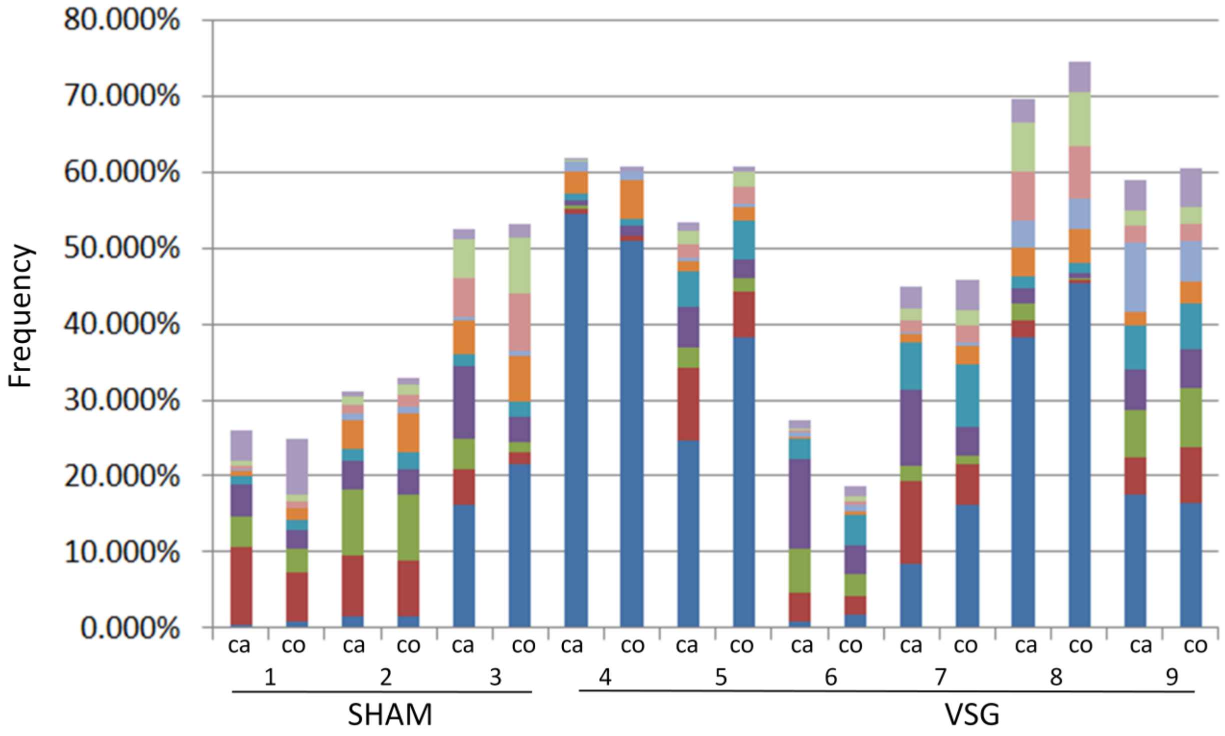

ESM Figure 6. Reproducibility of sequencing data from paired caecum (ca) and colon (co) samples from gastrectomised (vertical sleeve gastrectomy, VSG) Goto-Kakizaki rats and sham operated controls. rDNA motifs are color coded. Data are shown for the 10-most prevalent rDNA motifs across all samples.

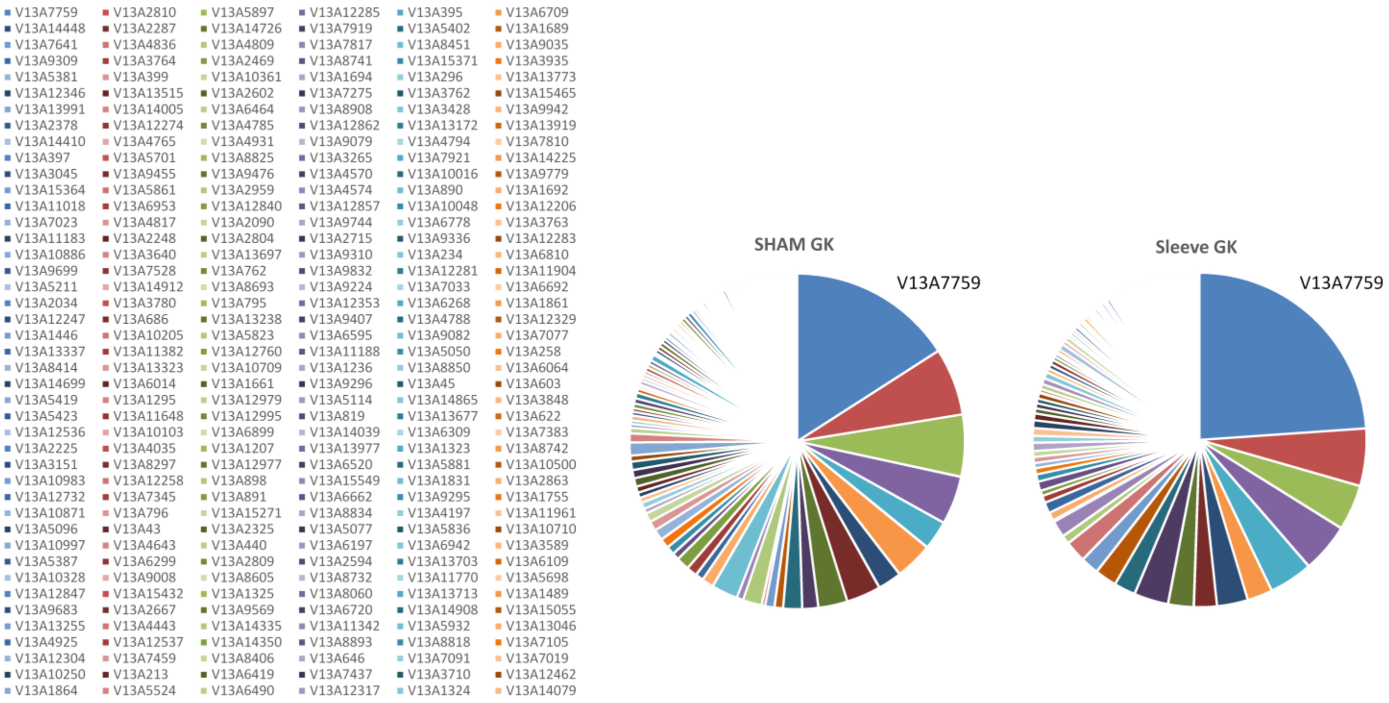

ESM Figure 7. Distribution of 16SrDNA motifs in gastrectomised (sleeve) Goto-Kakizaki (GK) rats and sham operated GK rats. Frequencies of the motifs acquired in caecum and colon samples in the two rat groups were merged. Sequences and frequencies of motifs in the two rat groups are given in Supplementary Table 2.

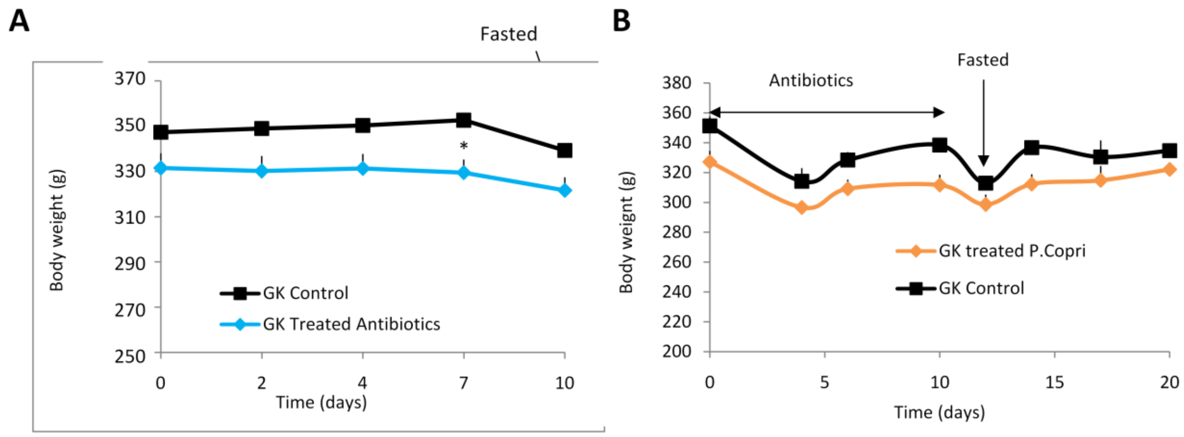

ESM Figure 8. Body weight follow up in Goto-Kakizaki (GK) rats following *Prevotella copri* enrichment. Rats were treated with an antibiotic cocktail allowing growth of *Prevotella copri* (A) or inoculated with either *Prevotella copri* or heat inactivated *Prevotella copri* (B).

*P<0.05 significantly different to GK controls.

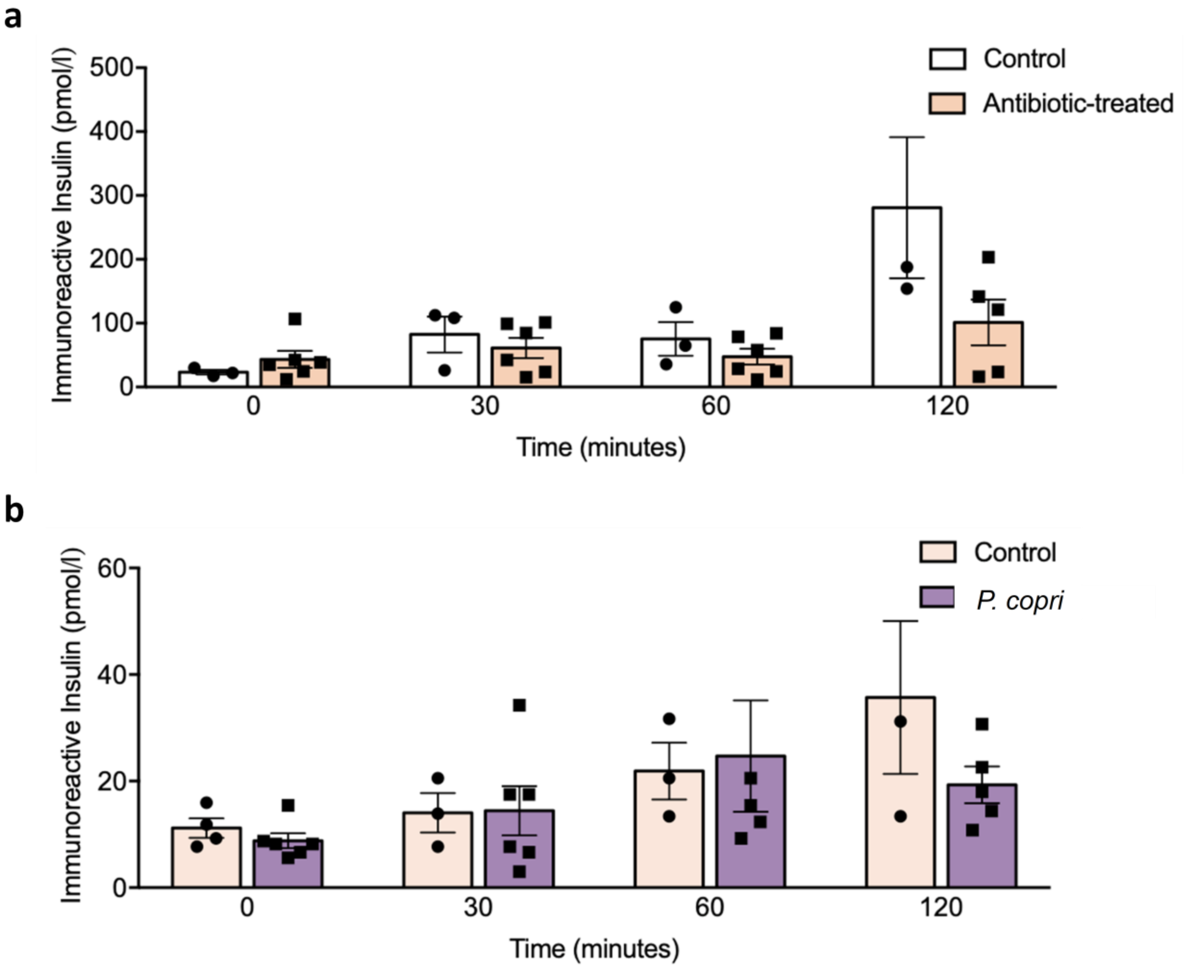

ESM Figure 9. Effects of gut microbiota enrichment in *Prevotella Copri* on insulin secretion in Goto-Kakizaki (GK) rats. Glucose-induced insulin secretion was determined in GK rats either treated with antibiotics permissive to *P. copri* (n=6) (a) or inoculated with *P. copri* (n=6) or heat inactivated *P. copri* (n=3) (b). Insulin secretion tests (see methods) were performed following an overnight (16h) fast 10 days after antibiotic treatment or *P. copri* inoculation. Data are mean ±SEM.

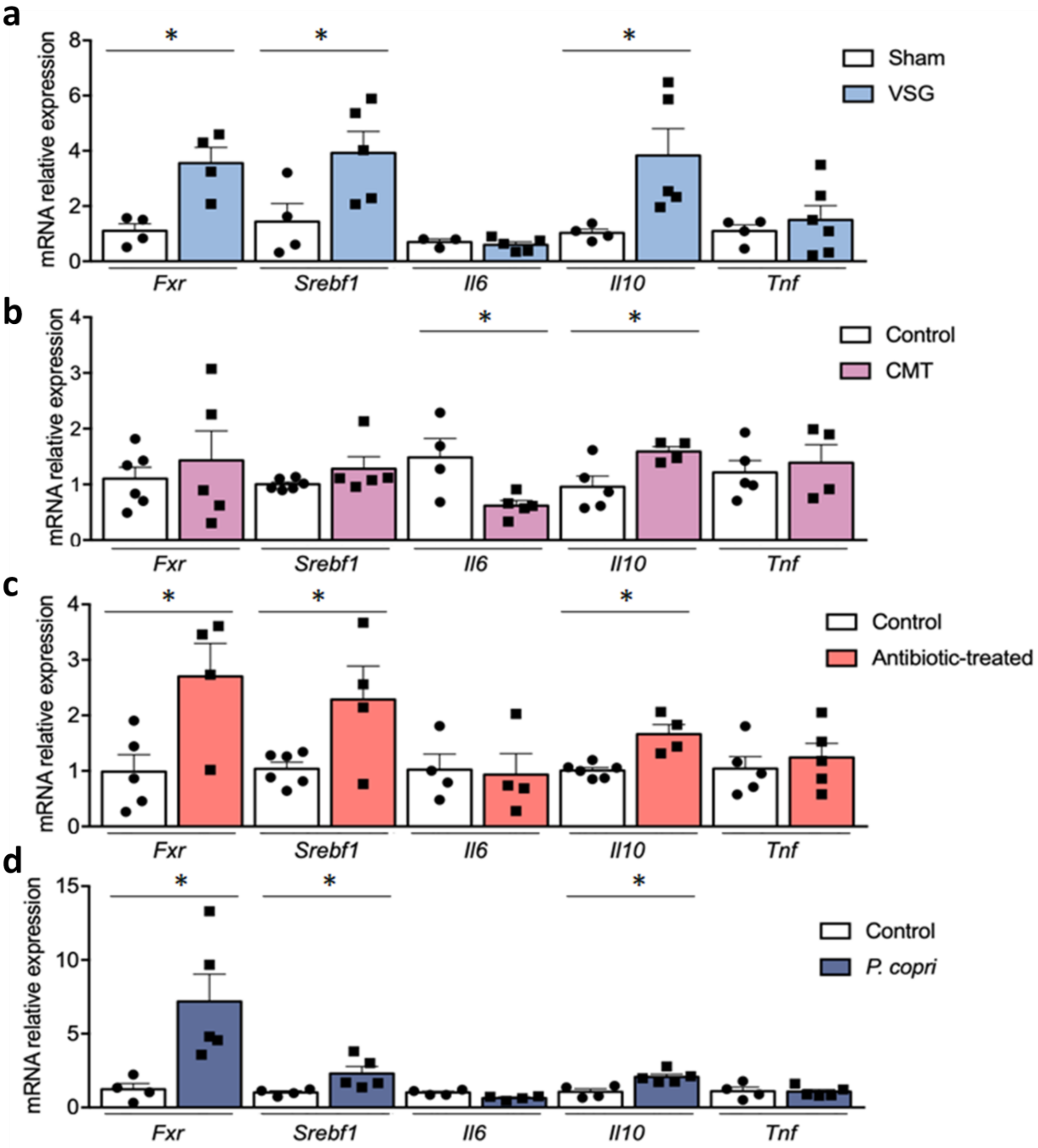

ESM Figure 10. Gene expression in adipose tissue in treated Goto-Kakizaki (GK) rats and controls. Gene expression assessed by quantitative RT PCR was determined in adipose tissue from GK rats following vertical sleeve gastrectomy (VSG) (a), caecal microbiota transfer (CMT) from VSG treated GK rats to GK rats (b), treatment of GK rats with *P. copri* permissive antibiotics (c) and inoculation of GK rats with *P. copri* (d). n= 4-6 per group. Data are mean ± SEM. *P<0.05 significantly different to control rats.
